# Institutionalization of deinstitutionalization: a cross-national analysis of mental health system reform

**DOI:** 10.1186/1752-4458-8-47

**Published:** 2014-11-22

**Authors:** Gordon C Shen, Lonnie R Snowden

**Affiliations:** School of Public Health, Yale University, 60 College Street, P.O. Box 208034, New Haven, CT 06520 USA; School of Public Health, University of California at Berkeley, 235 University Hall, Berkeley, CA 94720 USA

**Keywords:** Diffusion of innovation, Institutional theory, Governance, Mental health policy

## Abstract

**Background:**

Policies generate accountability in that they offer a standard against which government performance can be assessed. A central question of this study is whether ideological imprint left by policy is realized in the time following its adoption. National mental health policy expressly promotes the notion of deinstitutionalization, which mandates that individuals be cared for in the community rather than in institutional environments.

**Methods:**

We investigate whether mental health policy adoption induced a transformation in the structure of mental health systems, namely psychiatric beds, using panel data on 193 countries between 2001 and 2011.

**Results:**

Our striking regression results demonstrate that late-adopters of mental health policy are more likely to reduce psychiatric beds in mental hospitals and other biomedical settings than innovators, whereas they are less likely than non-adopters to reduce psychiatric beds in general hospitals.

**Conclusions:**

It can be inferred late adopters are motivated to implement deinstitutionalization for technical efficiency rather than social legitimacy reasons.

**Electronic supplementary material:**

The online version of this article (doi:10.1186/1752-4458-8-47) contains supplementary material, which is available to authorized users.

## Background

Countries that subscribe to international norms and ideas of progress and advancement uphold them in national health policies. Even when policies are ratified, national governments frequently fail to implement their terms and conditions. Why? Problems with implementing policies are especially pronounced due to institutional inertia, which is manifested in heated parliamentary deliberations and legislative proceedings. A whole host of other sociopolitical forces are at play during the implementation of health reform, such as cultural cleavages, resource availability, and the extent of political or legal infrastructure development. In this study, we are interested in whether deinstitutionalization policy galvanizes a revolution in the organization of national mental heath systems. Deinstitutionalization policy is a policy that mandates a shift in practice of caring for individuals with mental illness from institutional environments to the community. Institutionalization is a social process by which structures, policies, practices, and programs are instilled with enough value such that they first acquire social legitimacy, are normatively and cognitively held in place by members of the world society, become taken-for-granted by the collective, and ultimately achieve a “rule-like” status ([1–3]: 25, [4]). We argue that the institutionalization of deinstitutionalization policy is a two-fold process: isomorphism may be observed in the adoption of mental health policy across countries (first stage), but not necessarily in the make-up of state administrative apparatus and health care infrastructure (second stage) [5, 6]. Thus, the objective of this study is to empirically examine whether the institutionalization of deinstitutionalization policy changed the supply of psychiatric beds in 193 countries from 2001 to 2011^a^.

Public policies are broad statements of intentions and general directions their writers wish to undertake. They may also outline methods and principles that politicians, professional and industry groups, and other constituencies plan to use to achieve their directives. Policy statements, however, are not always complemented with local catchment area and organization plans, funding, programs, personnel, and regulations. Deinstitutionalization is a major, yet broad component of national mental health policies. The United Nations [7] and WHO [8] have both declared that mental health care should be shifted from hospital- to community-based treatment facilities. Deinstitutionalization is fundamentally an administrative philosophy rather than a technical advancement, so variances found in its implementation among countries invoke social legitimacy and cost-effectiveness impetuses behind policy change [9–11]. We test both the external legitimacy and internal efficiency hypotheses using the World Health Organization’s (WHO) *Mental Health Atlas*, a country-level panel dataset of mental health systems.

The pattern of policy diffusion reflets countries’ readiness for change and propensity to take political risks. Tracing the sigma-curve of innovation diffusion, a few early-adopters (“innovators”) are followed by a critical mass of late-adopters (“laggards”) and non-adopters (“resisters”) [12]. The phase of policy adoption lends itself as a predictor of mental health system change. Laggards are of particular interest to us because it is equally plausible for such countries to hold either a legitimacy or efficiency motivation in adopting deinstitutionalization. Institutional theorists assert that early adopters assume a certain organizational form because they are motivated by economic and technical needs, whereas late adopters conform because they are chiefly concerned with status enhancement [3, 13]. As such, actions of late-adopting countries reinforce the bandwagon effect because they are susceptible to norms institutionalized in the world society [14–16]. Proponents of the legitimacy side, however, often fail to recognize bureaucrats and technocrats’ ability to purposefully and creatively applying knowledge gained from earlier adopters [17]. With sufficient resources and stewardship, late adopters have the potential to implement a policy innovation such that efficiency gains are realized from policy adoption opportunities. Late-adopting countries could customize off the shelf policies so that treatment, preventive, and rehabilitation services can eventually be delivered at the mental health system’s optimal capacity.

This article is organized as follows. In the Theoretical background section we will review the relevant literature that support our interpretation of late-adopting nation-states’ behavior as being driven by internal efficiency or external legitimacy motivation. In the Methods section, we will give a brief overview on deinstitutionalization in the 21st century, then lay out our plan to test the two competing hypotheses using the WHO *Mental Health Atlas* and other secondary datasets. The results will be presented, followed by a discussion of the theoretical implications. We will conclude with a brief discussion of the policy implications.

### Theoretical background

#### Internal efficiency

Are mental health systems designed for efficiency or legitimacy reasons? Realists believe behavioral consistencies reflect inherent needs and interests. Rational choice theorists, in particular, consider nation-states as rational, unitary actors who are actualizing fixed preferences [18–20]. As such, there is a distinct economic rationale underlying policy adoption lag: policymakers valorize deinstitutionalization because it is instrumental in cutting the exorbitant cost of delivering mental health care in residential facilities and hospitals.

The mechanisms underlying policy adoption and implementation differ for early- versus late-adopters. Countries that are innovators in mental health care tend to face two dilemmas. Early on in the diffusion process, they face a lack of information: not all policy alternatives are known and the merits of the ones known are uncertain. The general lack of information about the cost of all policy options and the benefits of their concomitant solutions hampers governments’ ability to make rational decisions. Governments can only make predictions on the equity, quality, and efficiency implications of deinstitutionalization policy based on their own experience with reforming the general health sector.

Alternatively, early adopters with slack resources may invest them in experiments involving the reorganization of mental health system on a trial and error basis [21, 22]. Government stakeholders and special interest groups associated with the experiments have a large stake in their outcomes, so therein lies a chance that the test population of citizens are exploited in the process of carrying out the experiments [23, 24]. Another trade-off early adopters make once they embark on such an irreversible course of action is surrendering option value, or the benefit that incurs from delaying a decision to conduct experiments. High sunk costs are incurred if deinstitutionalization proves to be a failure because the political or financial price of reversing it is exceedingly high, or because the policy itself cannot be easily undone once enacted. On the upside, investment in pilots could pay off in dividends if pilot results are used to incrementally improve mental health systems. Pioneers in mental health care stand to reap the benefits of discovering new norms and practices in the form of increased technical know how and regional influence^b^.

Late adopters are risk averse and tend to learn from earlier adopters. The option value of decision-making is at its highest early on in the policy diffusion process, and so forbearance or waiting becomes the default strategy for decision makers concerned with risks [25]. Policymakers in late-adopting countries are indirectly affected by both the availability of and access to an evidence base of mental health policy’s effectiveness as it has been tested at home or abroad [12, 26–29]. To them, early-adopting jurisdictions are perceived to be laboratories, test sites, loci of experiments, pilots, and demonstration projects that produce technical information pertaining to an innovative policy [30, 31]. Policy-makers in late-adopting countries are thus the target audience of at least three types of research -clinical efficacy, cost-effectiveness, and policy research- produced in early-adopting jurisdictions [32]. Another complicating factor is access to scant evidence on deinstitutionalization nationally and globally; a 10/90 divide exists between developed and developing countries on accessibility to comparative studies on mental health services, programs, and policies [33, 34]. Any efforts taken to learn about natural experiments occurring elsewhere or pilots taking place at home would mediate the relationship between policy adoption and bed changes. This is a key factor believed to result in cross-national variation on the timing of policy adoption.

In sum, uncertainty and information asymmetry about mental health policy are expected to decrease over the life cycle of its adoption across countries [35, 36]. As countries gaining experience with deinstitutionalization continues to proliferate, its concomitant practices would spread through channels as diverse as international organizations, advocacy networks, consulting companies, academic meetings, dignitary visits and study tours ([37]: 367–368, [38]). Factual information on deinstitutionalization would not only serve as an aid in political debates and policymaking process, but also facilitate the adoption of a broad concept like deinstitutionalization to serve local needs, circumstances, and preferences. The option value and sunk cost both decrease over the adoption cycle, which suggests the following hypothesis:

***Hypothesis 1***: Late adopters of mental health policy are *more* likely than early adopters to gradually reduce the number of psychiatric beds within their country.

If we observe a steeper rate of institution-based care downsizing among late- than early-adopting countries, then we can infer that the former adopted mental health policy for internal efficiency reasons. More pointedly, if policy-makers ratified the deinstitutionalization component of mental health policy for efficiency reasons, then we expect a decrease in the number of psychiatric beds nationally during our decade-long study period^c^.

#### External legitimacy

Constructivists and realists have long disagreed on whether culture should be factored into the conceptual model of policy adoption and implementation. Constructivists challenge realists’ assumption of purposive rationality in organizational behavior. In Hall’s seminal study on the diffusion of Keynesian ideas, he noted, “the process whereby one policy paradigm comes to replace another is likely to be more sociological than scientific” ([39]: 280). Neo-institutional theorists, in particular, argue that organizations routinely follow taken-for-granted models, standards, and myths found in the institutional environment, regardless of their functional utility [1, 4]. Organizations gain legitimacy by incorporating elements of widely accepted cultural models into their structures and procedures [1, 40, 41]. Extrapolated to a macro level, countries are products of social norms, and so their action can be explained by logics of appropriateness in vogue in the world society [42, 43]. Ideas of the wider environment shape sovereign states’ social structures and regulatory behavior through implicit or explicit rules. And states help legitimate scripts in a cyclical manner following the neo-institutional argument^d^. Supranational institutions (e.g. WHO) and peer states independently or jointly influence a focal country’s behavior through models, standards, rules, and myths [40, 44–46]. As countries come to embody the same beliefs systems, their national identities are morphing, and their interests become shared through embedded exchanges with other actors in the international community. This socialization and habitualization process gives rise to a culture within the world society [46–48].

We invoke constructivist logic for the second foci of this study, which questions whether countries adopt mental health policy in order to enhance their legitimacy on the global stage. If this is the case, psychiatric beds would continue to accommodate patients in need of extended treatment even if those rotating in and out of these beds are better served by their families and in the community. Administrative structures created in response to technical demands have previously been demonstrated to be routinely decoupled from policy demands in education [49, 50], welfare [51] and human rights [52, 53]. The common explanation cutting across these domains is that policies were ratified as symbolic gestures. Policies thus “lose their bite,” or become obsolete, if the government is the sole actor in charge of policy implementation and no enforcement or accountability mechanisms are in place to monitor the extent of policy implementation [1, 35, 53, 54].

The phase of adoption poses as an additional consideration in our study of mental health policy implementation. Efficiency gains sought after by early adopters are gradually displaced by normative pressure to adopt isomorphic practices, forms, and policies among those remaining in the study population [1, 55]. Tolbert and Zucker found that early adopters of civil service reforms were motivated by technical or economic needs of city governments, whereas later adopters responded to the growing social legitimacy of these programs as taken-for-granted improvements to administrative apparatus [3]. Their hypothesis has since been empirically tested for the cases of adoption of personnel administration programs [56], CEO long-term incentive plans [57], Total Quality Management [58], drug abuse treatment units [59], and equal opportunity employment laws [60], What are the salient explanations arising from these studies? Late adopters can economize on search costs by imitating the actions of prior adopters [61]. They are less likely to conceive of a feasible way to actualize policy innovations, especially if they do not have the R&D (research and development) capacity. Finally, rapid spread of mental health reforms puts increasing pressure on laggards to jump on the bandwagon in order to avoid the stigma of appearing anachronistic [62–65]. We pose Tolbert and Zucker’s original hypothesis for deinstitutionalization practices across countries as follows:

***Hypothesis 2***: Late adopters of mental health policy are *less* likely than early adopters to gradually reduce the number of psychiatric beds within their country.

Our legitimacy hypothesis predicts that reduced variety would be observed in mental health policies, but variance would remain in the composition of mental health systems when comparing WHO member countries.

As the legitimacy of deinstitutionalization grows in the world polity, governments feel increasing pressure to comply with international norms and to ratify that particular component of mental health policy so as to not appear as a deviant country. Late adopters are more likely to treat deinstitutionalization as a social fact of health care reform rather than adopt it because it is compatible with intra-country circumstances [1, 3, 4, 66]. Decision makers in later adopting countries seek to enhance their legitimacy, credibility, and reputation by importing advanced policy innovations. To them, upholding a mental health policy could also act as a demonstration of modernity, shield for inaction, or veneer to cover up corruption ([53, 67]: 125). Adopting policy may additionally boost the public’s opinion and interest groups’ support of the national government, especially during an electoral cycle. The act of adoption itself, however, does not necessarily compel the national government to decrease the number of beds in psychiatric facilities, which is really the essence of deinstitutionalization. If so, late adopters would demonstrate no change in psychiatric bed rates during our decade-long study period compared to early adopters. The institutionalization of deinstitutionalization is rendered superficial if it stops short at policy adoption and does not penetrate any deeper into the transformation of mental health systems.

## Methods

### Context

The mental health sector is a fitting empirical context for us to test the two competing hypotheses about motivations behind policy adoption because the design of national mental health systems is subjected to strong functional demands and to principles legitimated in the world society [68, 69]. Deinstitutionalization is one of the major milestones in the care of people with mental, neurological, and substance use (MNS) disorders in the second half of the twentieth century. It is construed as an administrative apparatus that is designed to prevent chronic disability, uphold human rights, and reduce the cost of care [70].

Deinstitutionalization started gaining momentum in the 1950’s. As the deinstitutionalization process unfolded, however, policy planners and healthcare providers in North America and Western Europe began to realize the unanticipated consequences of this revolution in the mental health field. The deinstitutionalization movement is deemed successful if one focuses only on the benchmarks found in administrative datasets, typically used for reimbursement purposes, or in census of mental health facilities: closure of hospitals and asylums; cuts in the number of beds; decrease in rates of inpatient admission, bed rotation factor, average length of stay, and number of residents [71–74]. Yet, many countries continue to rely on mental hospitals as the main hubs of mental health care. Oftentimes they are not well maintained, resulting in patients having to live in squalid and deplorable conditions [75]. Psychiatric facilities may not even be fully equipped with medical equipment and basic amenities such as toilets, beds, and personal space. Staff-to-patient ratios are low in these facilities, partly owed to the global mental health workforce shortage, making it unlikely patients receive high quality care and individual attention [76]. Last, patients who reside in mental hospitals are segregated from society due to social stigma from the public, abandonment by their families, and the remote locations of hospitals.

Community-based care is considered to be more humane, higher quality and more cost-effective compared to institution-based care [77–79] ^e^. Custodial services provided by large institutions -most evidently mental hospitals and asylums- are only justified for a small proportion of patients with severe and chronic mental disorders. Patients with a protracted tenure in psychiatric facilities often have been there since signs and symptoms of their illness manifested during early childhood or adolescence [80–82] ^f^. Mental health care incurs exorbitant costs because of both the severity and chronicity of certain conditions. Patients of community-based treatment facilities fare better than patients of inpatient treatment on various outcome measures, such as relapse rate, delay or failure in help-seeking, treatment compliance, adherence to medication intake, number of admissions to inpatient or residential facilities, homelessness, illicit substance use, and criminal involvement [83, 84]. National governments are confronted with a two-fold challenge of managing this population’s chronic disorder and sustaining their livelihood in the community. The trick for governments is to find a judicious mix of community, outpatient, and inpatient services^g^.

The lack of synchronicity in closing or downsizing institution-based services with scaling-up community-based services has engendered a whole host of problems^h^. A sudden and abrupt reduction in psychiatric beds has resulted in the inadvertent transfer of patients to psychiatric units in general hospitals [85], nursing homes [86], households [87], supported housing [88], and prisons [89–91]. This is observed in the wake of “trans-institutionalization,” or the act of transferring patients from mental hospitals to other institutions such as homeless shelters, custodial institutions, and prisons [92, 93]. Recent research further suggests a nascent, reflexive trend in Europe towards the “re-institutionalization” of care, marked by the rising number of supported housing facilities, forensic psychiatric beds and penitentiaries (e.g. correctional facilities, jails, prisons), in addition to existing asylums, mental hospitals and private clinics [94–97]. The experiences of all early policy adopter countries converge on one salient point: if the process of deinstitutionalization unfolds too quickly, then the burden tend to shift to other human and human service sectors. Health and human service system simply were not ready to deal with servicer users who have heightened stress as a result of living in the community, let alone assist with their recovery process [98]. The lack of synchronicity between institution- and community-based services ultimately had collateral results of fragmentation of services, lack of quality assurance over available services, financial cutbacks, and workforce shortages [99].

Deinstitutionalization is a critical juncture from which vectors of mental health reform emerge^i^. Very few countries have achieved an optimal mix of mental health services just described. As a corollary, there is no gold standard for mental health system organization so that the needs of all people with MNS disorders are fulfilled. The trajectories of mental health care (re)organization offer researchers like us a provocative basis of comparison with respect to historical points of convergence or divergence for countries [48, 100, 101].

Developed and developing countries face different challenges when it comes to mental health system development. The population in developing countries make up 84% of the world’s population, and yet developing countries claim only 11% of the world’s net health spending [102]. Developing countries grapple with an under-provision of resources, personnel and services. Non-state actors working in them advocate for increased investment in those requiring mental health care. Governments of developing countries would counter these protests by saying that they are hard pressed to invest in trial-and-error experiments and search for the optimal mental health policy framework and implementation plan suitable for their population, especially under severe resource constraints. The situation is different for developed countries where the process of deinstitutionalization has led to closures of mental hospitals and asylums, as well as a reduction in the number of patients in the ones left standing. However, the development of community-based residential and occupational facilities and the uptake of incident clinical cases have not been commensurate with the pace of downsizing or closing psychiatric institutions [103]. Both developing and developed countries face problems such as parity in the provision of resources between physical and mental health services, the need to promote detection and treatment of mental disorders in primary care settings, and competing demands of psychiatric and other specialty services.

Since the deinstitutionalization movement among developed countries generally preceded the one among developing countries, the latter stands to gleam lessons from the former in three main respects: release of individuals from hospitals into the community; diversion from hospital admissions; and development of alternative community services [104]. This study addresses the second respect. We look specifically at changes in inpatient psychiatric bed rates among 193 countries over the course of a decade. Results of this study have implications for striking the proper mix of mental health service organizations and identifying the scope conditions under which deinstitutionalization happens.

### Data

The primary data source is the WHO *Mental Health Atlas* (“Atlas”). Atlas serves as a map of mental health infrastructure and resources in the world. A focal point for mental health in the Ministry of Health was responsible for completing the Atlas survey on behalf of his/her WHO member state, associate or area. In some instances the WHO regional offices assisted in collecting the data. Three waves of Atlas data are available: 2001 (n = 184), 2005 (n = 193), and 2011 (n = 184) [105–107].

### Dependent variable

The dependent variable should describe the physical capital of national mental health systems since we are interested in deinstitutionalization’s adoption to local contexts. The density of psychiatric beds, as enumerated from various biomedical settings, per country, and across time was selected as a fitting indicator for this purpose^j^. Hospital inpatient beds make up one of the most expensive components of mental health systems, accounting for up to three quarters of some national mental health budgets [108]. The questions of how many psychiatric beds are needed, how much they cost, and whether existing psychiatric beds are well managed and clinically appropriate continue to be heavily debated [109–115] ^k^. With that said, there are four potential indicators from Atlas related to beds: total number of psychiatric beds in the country; total number of beds in mental hospitals; total number of beds in general hospitals; and total number of beds in other settings. We struck the last candidate indicator from consideration because it was worded as “beds in other settings” for the 2001 and 2005 waves and as “beds in community residential facilities” in the 2011 wave. This inconsistency was confirmed through Spearman’s correlation comparing the pairs of cross-sectional data, which was high between 2001 and 2005 (rho = 0.96; p = 0.000), but low between 2001 with 2011 (rho = 0.42; p = 0.005) and between 2005 and 2011 (rho = 0.50; p = 0.001). This led us to question the consistency of rates of total beds across the three waves as well, but we found high Spearman correlations for the other three indicators. We proceeded with the analyses looking at population-scaled rates of total beds, general hospital beds, and mental hospital beds as outcomes of interest. We further transformed the three types of bed rates per 100,000 population into logarithms to control for outliers.

### Independent variables

The independent variable is the timing of deinstitutionalization adoption. The wording of the questions on national mental health policy was inconsistent across the three cross-sectional waves of Atlas: while the 2001 and 2005 waves asked about the existence of a national mental health policy and, if yes, the year of its *initial formulation*, the 2011 wave asked about the existence of an officially approved mental health policy and, if yes, the name of the document and the year of its *last revision*. To establish the earliest mental health policy adoption in each country, we cross-walked the Atlas data with data from two other datasets -the World Health Organization Assessment Instrument for Mental Health Systems (WHO-AIMS) and WHO MiNDbank- to verify whether and when each country had actually adopted it^l^. WHO-AIMS is a tool used to collect essential information on the mental health system of 42 low- and middle-income countries in 2005 [116, 117]. And WHO MiNDbank is an online platform for the sharing of information related to disability, human rights, mental health, health and development [118].

Deinstitutionalization is one specific component of national mental health policy [119]. Even though the first two waves of Atlas assessed the existence of five components in the national mental health policy, none were explicitly worded as deinstitutionalization^m^. In the absence of information about the attributes of mental health policies, the year of initial adoption of national mental health policy was taken as the main predictive indicator. There are three potential ways to treat the year of initial adoption of national mental health policy as an independent variable: nominal, ordinal, and continuous. The nominal variable is simply adopter versus non-adopter of any mental health policy. The ordinal variable indicates the five phases of adoption: innovators (2.5%; 1950–1959); early adopters (13.5%; 1960–1985); early majority (34%; 1986–1998); late majority (34%; 1999–2011); resisters or non-adopters (16%). Rogers [12] originally specified these cut-offs under a normal curve, which continues to be commonly used in policy diffusion research. Non-adopters and late majority make up the referent category of the nominal and ordinal variable, respectively. The year of mental health policy adoption was also treated as a continuous variable, which was zeroed on the year before the first historic adoption in the WHO. We specified models using one of the three functional forms as a robustness check of the assumption of a linear relationship between mental health policy adoption and bed rate change. The independent and control variables were all lagged by one year. This way, the risk of adoption in each year depends on the characteristics of the sampled countries in the prior calendar year. The lag effects are intended to address simultaneity bias; otherwise, if lag effects are not used, regression coefficients will be overestimated and the standard errors will be underestimated.

### Control variables

#### Mental health system characteristics

Control variables are characteristics that could moderate the relationship between mental health policy adoption and implementation. We included a number of mental health system and country characteristics as control variables in our analysis for this reason. Mental health policy has greater effectiveness when it is accompanied by a mental health plan or law since they help translate the vision, values and principles articulated in policy into concrete strategies and activities [120, 121] ^n^. The year of initial formulation of national mental health plan and law were thus controlled for in our analysis.

Formal and informal human resources are the front line workers who deliver mental health services. The mental health workforce is a channel that facilitates the exchange of ideas and knowledge across geopolitical borders [44, 76]. Ruef and Scott [122] have delineated this so-called “normative isomorphic” force into managerial legitimacy (e.g. efficiency, cost-containment) and technical legitimacy (e.g. patient care quality, specialty training). The authority of health care professionals is derived from their ability to develop and translate rationalized and universalistic knowledge ([123, 124]: 24). Professional associations, such as the World Psychiatric Association, hold conferences and issue guidelines and newsletters to uphold clinical standards. Health care professionals are also in the position to provide expert advice and recommendations to administrators and policymakers, so their stance on deinstitutionalization can either spur or thwart the movement on a national or global level. Taken together, mental health professional presence is operationalized as the logged rates of psychiatrists, nurses, psychologists, and social workers per 100,000 population in our analysis.

The notion of recovery has permeated advocacy efforts to promote mental health care in the community. Recovery neatly couples with deinstitutionalization in that it entails non-coercive therapeutic alliances between professional and service users, autonomy among service users, and empowerment on the part of users and their families [125–127]. Civil society advocates for selected policy ideas, inculcate awareness of deinstitutionalization to the public, and generally promote certain policy cues [128]. International NGOs (INGOs), such as the World Network of Users and Survivors of Psychiatry, World Federation of Mental Health, and MindFreedom International, and local NGOs, such as Basic Needs in the UK, Mental Disability Advocacy Centre in Budapest, and Disability Rights International in the US, are champions of recovery and other missing or neglected elements in existing mental health policies [129, 130]. INGOs maintain contact networks through which ideas and discourses are spread across geopolitical borders [131–134]. Together with local NGO’s, they demand corrective actions from governments and mental health professionals. Local user and family associations are also well positioned to advocate for families as primary caretakers of patients. The organizational structure of voluntary associations mentioned are more nimble and adaptive than government agencies and professional associations, so they are predisposed to reacting quickly to environmental exigencies. We thus operationalized interest group presence as two dichotomous variables: the existence of at least one user or family association and NGOs’ involvement in mental health the same country.

Deinstitutionalization efforts would ideally be tailored to the availability of financial resources [135, 136]. A policy innovation could either stimulate huge appropriations or have little monetary impact, depending on the fiscal conditions under which its adoption occurred [31]. If slack resources are available, then decision makers can afford the luxury to experiment and to accept the risk of potential failure [61]. Investment is an explicit, observable, and irrevocable proxy of a focal government’s commitment to a mental health policy innovation [137, 138] ^o^. Dedicated and ongoing funding to implement and routinize deinstitutionalization is thus operationalized as the total expenditure on health as a percentage of GDP, which varies by country-year. We collected these figures from the WHO National Health Account and the World Bank’s World Development Indicators databases.

Five other features of mental health systems were taken into account. The magnitude of the mental health policy problem is captured by annual prevalence rates of MNS disorders on the country level, with international epidemiological data for all three waves provided by the Global Burden of Disease Study [139–143] ^p^. Specifically, the logged rate of disability adjusted life-years due to MNS disorder per 100,000 population was included in our analysis. Information on the scale of MNS disorders could help determine resource allocation and development priorities by ministries of health and finance [144]. The usage of an information system to keep track of the transition from institution- to community-based care was therefore controlled for in our analyses. The caveat here is that application of the same data in monitoring and evaluating mental health systems was not assessed in Atlas.

Mental health problems often co-occur with acute and chronic physical health problems. Furthermore, access to mental health specialists is challenging even though effective treatment exists for the most common mental health problems and their comorbid conditions. The integration of mental health care into primary care and community-based settings is beneficial in addressing these problems. They improve access to mental health care, provide care for comorbid physical health problems in order improve health outcomes holistically, avoid of fragmentation of health services; reduce social stigma, and optimize the small number of psychiatric specialists [145–149]. A viable, pragmatic way to integrate mental health into primary health care or community-settings is task-shifting, or having specialists transfer some of their clinical skills to non-specialists typically through classroom training, followed by clinical supervision. Such integration programs would ideally be in place in order for individuals with common mental health problems to thrive in the community. To determine if clinical consultations have been held outside of psychiatric institutions, we controlled for the integration of mental health into general health care and the existence of a system of community-based care.

A segment of that same population requires integrated therapy consisting of psychosocial and pharmacological interventions. The discovery of antipsychotic medication has been credited to complement the development of community-based psychosocial treatment and rehabilitation [82, 150]. And antidepressant and antipsychotic drugs are typically listed on national essential drug lists. Therefore, we controlled for national expenditure on this particular consumable, as a percentage of total expenditure on health, in our analysis.

### Country characteristics

Certain country-level factors can also enable or inhibit mental health policy implementation. We included six factors that are motivated by prior literature. Governments face difficult choices in prioritizing mental health over other issues, especially in the midst of a global economic downturn. In addition to health expenditure as a percentage of GDP, we used the sampled countries’ income category to see if it affects psychiatric bed rates. The World Bank classifies countries according to 2012 GNI per capita in US dollars using the World Bank Atlas method, thus yielding low, lower middle, upper middle, and high income economies [151]. These four groups were included in our analysis as an ordinal variable.

Disasters, devastating in and of themselves, present an opportunity for radical innovation within the mental health system. Natural and technological disasters disrupt the order of a country’s health system, and could potentially spur changes in the quantity of beds during the rebuilding efforts. We controlled for the annual count of the disasters, which was furnished by the International Disaster Database [152]. Man-made disasters are anticipated shocks to countries engaged in them. During times of war, governments are more likely to allocate resources on national defense rather than on other policy agenda items. For this reason, we used data on the number of historical intra-, inter-, and extra-state wars from the Correlates of War Project [153, 154]. A dichotomous variable for any engagement in war and a count variable of the number of wars in a given year were included in our analysis. The results would shed light on whether deinstitutionalization is part of the transition governments made so that the emergency state would be on a more sustainable footing.

Mental health policy formulation and implementation are directly tied to government effectiveness, so we included a measure from the World Bank’s Governance Matters Project. Governments that are coopted by elite groups in society are less likely to enforce policies benefitting the disenfranchised, namely those with MNS disorders. We statistically controlled for the potential negative effects of ethnic, linguistics, and religious cleavages on mental health policy implementation using Fractionalization Data [155]. This summary score and its three components -ethnic, linguistic, language- were separately controlled for in our analysis. And finally, governments prioritize the needs, rights, and interests of people with MNS disorders to varying degrees. To test this claim, we included the proxy of Physical Integrity Right Index from the Cingranelli-Richards Human Rights Dataset in our models [156, 157]. Political imprisonment, a dimension of physical integrity, was tested separately in our analysis because psychiatric institutionalization has been used as an instrument of political control and social oppression.

Selected covariates were log transformed and centered to avoid potential collinearity problems where we observed outliers from their scatter plots against the dependent variable. For variables with multiple indicators, we performed sensitivity analyses to explore the degree of correlation between indicators as initial evidence of reliability. We also fitted separate models for candidate indicators. All control variables described are summarized in Additional file 1.

### Statistical model

The analysis entails running random effects (RE) linear models according to the following prediction equation:


where Y_it_ represents the logged rate of psychiatric beds, β’s are the matrices of parameter estimates, *i* represents country and *t* is the year of observation subscript, and ϵ_it_ and μ_t_ are the respective between- and within-period error terms. Regional-level factors cause errors to be correlated across observations, or intra-cluster correlation, so we chose RE modeling as an estimation approach to produce efficient estimates. In RE models, the variation across countries, or psu’s, is assumed to be random and uncorrelated with the main predictor or the other independent variables included in the model. The assumption behind RE is that the error term is not correlated with predictor variables, thus allowing time-invariant, country-specific characteristics to be explanatory variables. In other words, the RE model assumes that the intercepts differ for each country, or corr (μ_t_, MHPol_it_) = 0.

We compared RE models to log linear and fixed effects (FE) linear models for sensitivity analysis. RE models were compared to log linear models using the Breusch-Pagan (B-P) Lagrange multiplier test. If the B-P null hypothesis is rejected, then RE is preferred. If we fail to reject the B-P null hypothesis, then log linear is appropriate because no clustering is observed across the three waves. FE modeling was used as the alternative estimation approach to address potential omitted variable bias problems with RE models, which cause the error terms to be correlated with the independent variables. FE models remove the effect of these omitted, time-invariant characteristics from the predictor variables so the predictors’ effect could be better assessed. Like RE, the FE model accounted for clustering in the data by estimating a separate intercept for each wave while the log linear regression model estimated a common intercept for all countries in the sample. We used the Hausman test to compare the RE and FE models. If the Hausman null hypothesis is rejected then we will use the FE model because it is more consistent, whereas if we fail to reject the Hausman null then the RE model is retained because it is more efficient.

The three sets of aforementioned models were produced with only the independent variable (e.g. MHPol_it_). We then repeated the procedure for the multivariate analysis with independent and control variables. Log linear models were also produced for each wave, and goodness-of-fit chi-square test was used to see how well each model fit the data. The FE and RE models included wave-specific intercepts, and the robust option was used to correct for heteroskedasticity. Stata version 12 was used for all analyses.

## Results

**Figure 1:**
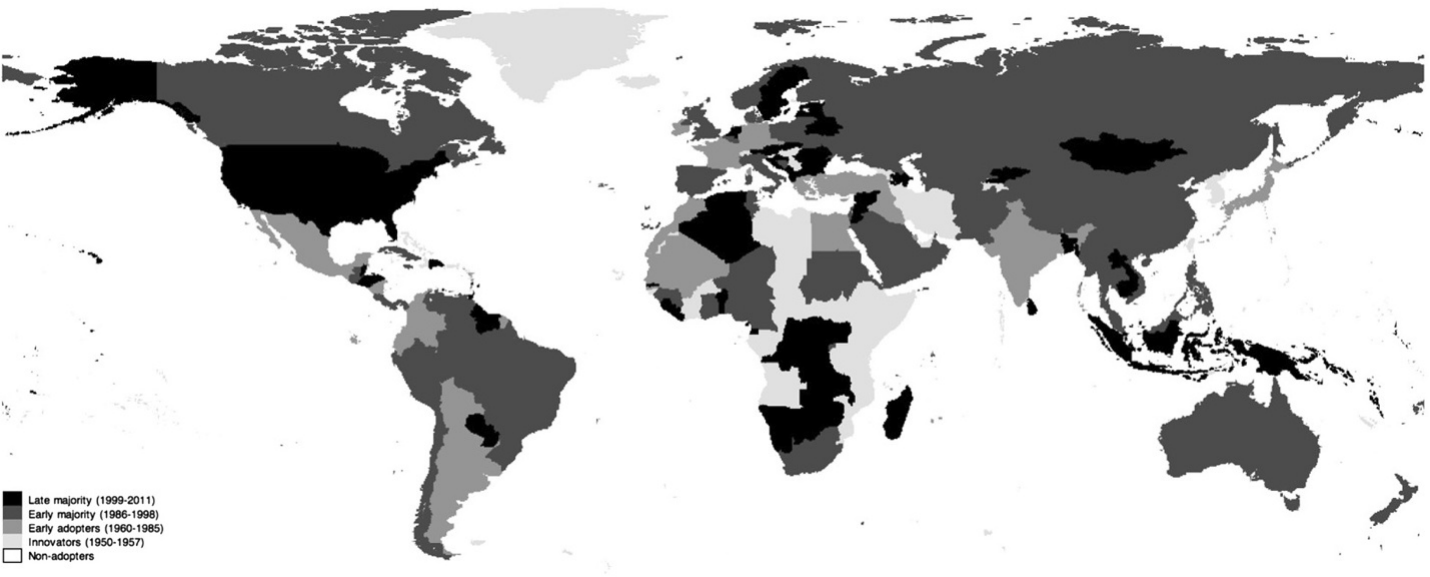
**Phases of mental health policy adoption.**

Atlas indicates that 148 countries adopted mental health policy and 45 did not from 1950 to 2011. Figure 1 is a map showing countries in various stages of mental health policy adoption. Descriptive statistics and correlation matrices for mental hospital beds, general hospital beds, and all psychiatric beds are respectively presented in Tables 1, 2, and 3. Univariate regression results for the main independent variable are reported in Table 4. To assess the relationship between phase of mental health policy adoption and bed rates we had initially divided the ordinal variable into five categories: innovators, early adopters, early majority, late majority, and resisters. Results of Table 4 show that between phase effects were almost entirely driven by the innovators, pointing to a stark difference between innovators and other adoption groups in mental health system reform. Robustness checks, not shown but available upon request by the authors, further show that between phase effects were nullified once we combined innovators and early adopters into a single category and compared them to late-adopters (referent category). We ultimately retained the ‘innovators’ category, collapsed the three later adopting groups (early adopter, early majority, late majority) into a ‘late adopters’ category, and renamed resisters as ‘non-adopters’ for subsequent analyses because innovators have played a unique role in mental health policy diffusion [3, 12, 39]. Regression results for the final linear random effects models are reported in Table 5. Models 1, 3, and 5 (“baseline models”) include only control variables, while models 2, 4, and 6 (“multivariate models”) contain independent and control variables for mental hospital bed rates, general hospital bed rates, and overall bed rates, respectively.

**Table 1:** **Descriptive statistics and pairwise correlation matrix of all psychiatric beds**

Variable	Mean	S.D.	Min	Max	1	2	3	4	5	6	7	8	9	10	11	12	13	14	15	16	17	18	19	20	21
1. Log of all psychiatric beds	2.76	1.71	-4.05	5.76																					
2. Mental health policy	1.58	1.51	0	4	-0.01																				
3. Mental health plan	2.78	1.35	0	4	-0.06	-0.45*																			
4. Mental health law	2.48	1.47	0	4	0.45*	-0.17	0.03																		
5. Log of psychiatrists	0.01	2.12	-6.21	3.72	0.83*	0.02	-0.05	0.45*																	
6. Log of nurses	1.05	2.32	-6.91	6.81	0.79*	-0.04	-0.02	0.41*	0.77*																
7. Log of psychologist	-0.32	2.4	-6.21	4.66	0.65*	0.00	0.01	0.30*	0.77*	0.63*															
8. Log of social workers	-0.55	2.47	-6.61	6.17	0.64*	0.06	-0.02	0.28*	0.71*	0.63*	0.80*														
9. Mental-primary care	0.93	0.26	0	1	0.29*	-0.12*	0.03	0.12*	0.20*	0.23*	0.16*	0.05													
10. Community care	0.76	0.42	0	1	0.25*	0.03	-0.05	0.15*	0.24*	0.30*	0.18*	0.19	0.32												
11. User & family assocs.	0.79	0.41	0	1	0.15*	-0.08	-0.08*	0.24*	0.20*	0.08	0.18*	0.10*	0.11*	0.08											
12. NGO’s	0.88	0.32	0	1	0.06	-0.10*	0.06	0.07	0.04	0.13*	-0.04	0.13*	0.24*	0.24*											
13. Health info.tech.	1.22	0.47	0	2	-0.16*	0.03	-0.04	-0.10*	-0.17*	-0.22*	-0.04	-0.04	-0.19*	-0.33*	-0.01	-0.03									
14. Log of MNS disorders	8.03	0.37	5.31	8.68	0.29*	-0.06	-0.07	0.17*	0.33*	0.31*	0.26*	0.13*	0.13*	0.30*	0.01	-0.01	-0.31*								
15. Pharmaceutical; Health spending	23.12	10.44	6	70.5	-0.30*	0.15*	0.00	-0.21*	-0.34*	-0.30*	-0.32*	-0.09*	-0.18*	-0.09*	-0.04	-0.07	-0.13*								
16. Health spending (%GDP)	6.42	2.75	0.5	21.89	0.26*	-0.01	0.08	0.24*	0.34*	0.28	0.45*	0.32*	0.12*	0.12*	0.21*	0.11*	-0.13*	0.22*	-0.20*						
17. World Bank income group	2.35	1.11	1	4	0.69*	0.10*	-0.05	0.29*	0.77*	0.67*	0.73*	0.71*	0.18*	0.35*	0.13*	0.08	-0.19*	0.32*	-0.34*	0.24*					
18. Log of disasters	1.13	0.96	0	4.56	-0.26*	-0.05	-0.03	-0.03	-0.08	-0.17*	-0.117*	-0.13*	0.01	-0.02	0.17*	0.15*	0.07	-0.01	0.08	-0.01*	-0.12*				
19. Instances of war	0.12	0.33	0	1	-0.24*	-0.08	0.09	0.03	-0.11*	-0.09	-0.08	0.04	-0.07	-0.05	0.14*	-0.01	0.00	-0.06	-0.02	0.01	-0.03	0.30*			
20. Ethnolinguistic fractionalization	1.24	0.60	0.01	2.52	-0.33*	-0.04	0.03	-0.17*	-0.48*	-0.29*	-0.43*	-0.33*	-0.11*	-0.12*	-0.04	-0.05	0.05	-0.20*	0.06	-0.17	-0.35*	0.07	0.15*		
21. Political imprisonment	1.22	0.83	0	2	0.35*	0.06	-0.02	0.15*	0.36*	0.36*	0.46*	0.34*	0.02	0.12*	0.05	0.14*	-0.06	0.14*	-0.15*	0.31*	0.39*	-0.29*	-0.11*	-0.14*	
22. Government effectiveness	-0.08	0.99	-2.32	2.34	0.57*	-0.01	0.07	0.31*	0.69*	0.62*	0.66*	0.69*	0.21*	0.35*	0.19*	0.17*	-0.14*	0.25*	0.30*	0.30*	0.18*	-0.81*	0.02	-0.27*	0.40*

*p < 0.05.

**Table 2:** **Descriptive statistics and pairwise correlation matrix of psychiatric beds in mental hospitals**

Variable	Mean	S.D.	Min	Max	1	2	3	4	5	6	7	8	9	10	11	12	13	14	15	16	17	18	19	20	21
1. Log of all mental hospital beds	2.54	1.7	-3.51	5.44																					
2. Mental health policy	1.58	1.51	0	4	-0.04																				
3. Mental health plan	2.78	1.35	0	4	-0.10*	-0.45*																			
4. Mental health law	2.48	1.47	0	4	0.42*	-0.17*	0.03																		
5. Log of psychiatrists	0.01	2.12	-6.21	3.72	0.79*	0.02	-0.05	0.45*																	
6. Log of nurses	1.05	2.32	-6.91	6.81	0.76*	-0.04	-0.02	0.41*	0.77*																
7. Log of psychologists	-0.32	2.4	-6.21	4.66	0.61*	0.00	0.01	0.3*	0.77*	0.63*															
8. Log of social workers	-0.55	2.47	-6.91	6.17	0.62*	0.06	-0.02	0.28	0.71*	0.62*	0.80														
9. Mental-primary care	0.93	0.26	0	1	0.22*	-0.12*	0.03	0.12*	0.20*	0.23*	0.16*	0.05*													
10. Community care	0.76	0.42	0	1	0.22*	0.03	-0.05	0.15*	0.24*	0.30*	0.18*	0.19	0.32*												
11. User & family assocs.	0.79	0.41	0	1	0.001	-0.08	0.16*	0.24*	0.20*	0.08	0.18*	0.10	0.11*	0.08											
12. NGO’s	0.88	0.32	0	1	0.04	-0.10*	0.06	0.07	0.04	0.13*	-0.02	-0.04	0.13*	0.24*	0.24*										
13. Health info. tech.	1.12	0.47	0	2	-0.13*	0.03	-0.04	-0.1*	-0.7*	-0.22*	-0.04	-0.04	-0.19*	-0.33*	-0.01	-0.03									
14. Log of MNS disorders	8.03	0.37	5.31	8.68	0.26*	-0.06	-0.07	0.17*	0.33*	0.31*	0.26*	0.13*	0.13*	0.30*	0.01	-0.01	-0.31*								
15. Pharmaceutical: Health spending	23.12	10.44	6	70.5	-0.26*	0.15*	0.00	-0.21*	-0.34*	-0.32*	-0.32*	-0.32*	-0.09*	-018*	-0.09*	-0.04	0.07	-0.13*							
16. Health spending (%GDP)	6.42	2.75	0.5	21.89	0.26*	-0.01	0.08	0.24*	0.34*	0.28*	0.45*	0.32*	0.12*	0.12*	0.21*	0.11*	-0.13*	0.22*	-0.20*						
17. World Bank income group	2.35	1.11	1	4	0.63*	0.10*	-0.05	0.29*	0.77*	0.67*	0.73*	0.71*	0.18*	0.35*	0.13*	0.08	-0.19*	0.32*	-0.34*	0.24*					
18. Log of disasters	1.13	0.96	0	4.56	-0.26*	-0.05	-0.03	-0.03	-0.08	-0.17*	-0.17*	-0.13*	0.01	-0.02	0.17*	0.15*	0.07	-0.01	0.08	-0.1*	-0.12*				
19. Instances of war	0.12	0.33	0	1	-0.24*	-0.08	0.09	0.03	-0.11*	-0.09	-0.08	0.04	-0.07	-0.05	0.14*	-0.01	0.00	-0.06	-0.02	0.01	-0.03	0.30*			
20. Ethnolinguistic fractionalization	1.24	0.6	0.01	2.52	-0.33*	-0.04	0.03	-0.17*	-0.48*	-0.29*	-0.43*	-0.33*	-0.11*	-0.12*	-0.04	-0.05	0.05	-0.20*	0.06	-0.17*	-0.35*	0.07	0.15*		
21. Political imprisonment	1.22	0.83	0	2	0.35*	0.06	-0.02	0.15*	0.36*	0.36*	0.46*	0.34*	0.02	0.12*	0.05	0.14*	-0.06	0.14*	-0.15*	0.31*	0.39*	-0.29*	-0.11*	-0.14*	
22. Government effectiveness	-0.08	0.99	-2.32	2.34	0.57*	-0.01	0.07	0.31*	0.69*	0.62*	0.69*	0.66*	0.21*	0.35*	0.19*	0.17*	-0.14*	0.25*	-0.30*	0.30*	0.81*	-0.05	0.02	-0.27*	0.40*

*P < 0.05.

**Table 3:** **Descriptive statistics and pairwise correlation matrix of psychiatric beds in general hospitals**

Variable	Mean	S.D.	Min	Max	1	2	3	4	5	6	7	8	9	10	11	12	13	14	15	16	17	18	19	20	21
1. Log of all general hospital beds	0.98	1.84	-4.96	5.25																					
2. Mental health policy	1.58	1.51	0	4	0.14*																				
3. Mental health plan	2.78	1.35	0	4	-0.17*	-0.45*																			
4. Mental health law	2.48	1.47	0	4	0.35*	-0.17	0.03																		
5. Log of psychiatrists	0.01	2.12	-6.21	3.72	0.71*	0.02	-0.05	0.45*																	
6. Log of nurses	1.05	2.32	-6.91	6.81	0.63*	-0.04	-0.02	0.41*	0.77*																
7. Log of psychologists	-0.32	2.4	-6.21	4.66	0.66*	0.00	0.01	0.30*	0.77*	0.63*															
8. Log of social workers	-0.55	2.47	-6.91	6.17	0.60*	0.06	-0.02	0.28*	0.71*	0.62*	0.80*														
9. Mental-primary care	0.93	0.26	0	1	0.15*	-0.12*	0.03	0.12*	0.2*	0.23*	0.16*	0.05													
10. Community care	0.76	0.42	0	1	0.19*	0.03	-0.05	0.15*	0.24*	0.3*	0.18*	0.19*	0.32*												
11. User & family assocs.	0.79	0.41	0	1	0.13*	-0.08	0.16*	0.24*	0.20*	0.08*	0.18*	0.10	0.11*	0.08											
12. NGO’s	0.88	0.32	0	1	0.00	-0.10*	0.06	0.07	0.04	0.13*	-0.02	-0.04	0.13*	0.24*	0.24*										
13. Health in.tech	1.12	0.47	0	2	-0.16*	0.03	-0.04	-0.10*	-0.17*	-0.22*	-0.04	-0.04	-0.19*	-0.33*	-0.01	-0.03									
14. Log of MNS disorders	8.03	0.37	5.31	8.68	0.23*	-0.06	-0.07	0.17*	0.33*	0.26*	0.13*	0.13*	0.30*	0.01	-0.01	-0.31*									
15. Pharmaceutical;; Health spending	23.12	10.44	6	70.5	-0.36*	0.15*	0.00	-0.21*	-0.34*	-0.32*	-0.32*	-0.09*	-0.18*	-0.09*	-0.04	0.07	-0.13*								
16. Health spending (% GDP)	6.42	2.75	0.5	21.89	0.41*	-0.01	0.08	0.24*	0.34*	0.28*	0.45*	0.32*	0.12*	0.12*	0.21*	-0.11*	-0.13*	0.22*	-0.20*						
17. World Bank income group	2.35	1.11	1	4	0.64*	0.10*	-0.05	0.29*	0.77*	0.67*	0.73*	0.71*	0.18*	0.35*	0.13*	0.08*	-0.19*	0.32*	-0.34*	0.24*					
18. Log of disasters	1.13	0.96	0	4.56	-0.18*	-0.05	-0.03	-0.03	-0.08	-0.17*	-0.17*	-0.13*	0.01	-0.02	0.17*	0.15*	0.07	-0.01	0.08	-0.10	-0.12*				
19. Instances of war	0.12	0.33	0	1	-0.12	-0.08	0.09	0.03	-0.11*	-0.09	-0.07	0.04	-0.07	-0.05	0.14*	-0.01	0.00	-0.06	-0.02	0.01	-0.03	0.30*			
20. Ethnolinguistic fractionalization	1.24	0.6	0.01	2.52	-0.34*	-0.04	0.03	-0.17*	-0.48*	-0.29*	-0.43*	-0.33*	-0.11*	-0.2*	-0.04	-0.05	0.05	-0.20*	0.06	-0.17*	-0.35*	0.07	0.15		
21. Political imprisonment	1.22	0.83	0	2	0.46*	0.06	-0.02	0.15*	0.36*	0.36*	0.46*	0.34*	0.02	0.12*	0.05	0.14*	-0.06	0.14*	-0.15*	0.31*	0.39*	-0.29*	-0.11*	0.14	
22. Government effectiveness	-0.08	0.99	-2.32	2.34	0.63*	-0.01	0.07	0.31*	0.69*	0.62*	0.69*	0.66*	0.21*	0.35*	0.19*	0.17*	-0.14*	0.25*	-0.30*	0.30*	0.81*	-0.05	0.02	0.27	0.40*

*p < 0.05.

**Table 4:** **Univariate linear random effects models predicting psychiatric bed rates** ^**a**^

	Psychiatric beds in mental hospitals	Psychiatric beds in general hospitals	Psychiatric beds in all settings
Innovators	1.60***	1.39	1.83***
	(0.39)	(0.82)	(0.42)
Early adopters	-0.24	0.21	0.04
	(0.38)	(0.43)	(0.38)
Early majority	-0.13	0.13	0.29
	(0.32)	(0.34)	(0.32)
Non-adopters	-0.48	0.63	-0.09
	(0.45)	(0.44)	(0.38)
Constant	2.60***	0.71**	2.62***
	(0.23)	(0.24)	(0.25)
Number of observations	430	408	457

*p < 0.05, **p < 0.01, ***p < 0.001.

^a^Robust standard errors are in parentheses. Late majority is the reference group for the mental health policy adoption variable.

**Table 5:** **Multivariate linear random effects models predicting psychiatric bed rates** ^b^

Independent variable	1	2	3	4	5	6
Innovators		1.23*		0.51		1.09*
		(0.37)		(0.97)		(0.25)
Non-adopters		-0.09		1.25*		-0.02
		(0.41)		(0.34)		(0.30)
Mental health plan	-0.02	0.00	-0.28*	-0.16	-0.07	-0.05
	(0.08)	(0.09)	(0.12)	(0.12)	(0.07)	(0.08)
Mental health law	0.15	0.16	0.17	0.19	0.20*	0.20*
	(0.10)	(0.11)	(0.12)	(0.12)	(0.08)	(0.08)
Log of psychiatrists	0.27*	0.26*	0.23+	0.23+	0.25*	0.25*
	(0.11)	(0.11)	(0.12)	(0.12)	(0.08)	(0.08)
Log of nurses	0.27*	0.26*	0.21*	0.23*	0.25*	0.25*
	(0.06)	(0.06)	(0.09)	(0.09)	(0.05)	(0.05)
Log of psychologists	-0.03	-0.02	0.16	0.15	-0.08	-0.07
	(0.09)	(0.09)	(0.17)	(0.17)	(0.07)	(0.07)
Log of social workers	0.02	0.01	-0.15	-0.14	0.03	0.03
	(0.07)	(0.07)	(0.13)	(0.13)	(0.06)	(0.06)
Mental-primary care	1.36+	1.43+	-0.60	-0.63	0.61	0.67
	(0.76)	(0.76)	(0.99)	(0.96)	(0.42)	(0.42)
Community care	-0.05	-0.06	-0.20	-0.18	0.06	0.04
	(0.15)	(0.16)	(0.26)	(0.26)	(0.13)	(0.13)
User and family assocs	-0.40	-0.48	-0.41	-0.32	-0.41	-0.47
	(0.35)	(0.37)	(0.42)	(0.39)	(0.31)	(0.32)
NGO’s	-0.01	-0.04	0.13	0.87	0.17	0.18
	(0.42)	(0.53)	(0.78)	(0.66)	(0.44)	(0.50)
Health info. tech.	0.16+	0.18+	-0.03	-0.05	0.05	0.06
	(0.09)	(0.09)	(0.13)	(0.13)	(0.07)	(0.07)
Log of MNS disorders	0.10	0.09	0.13	0.16	0.05	0.05
	(0.09)	(0.09)	(0.14)	(0.14)	(0.07)	(0.07)
Pharmaceutical:Health spending	-0.02	-0.02	-0.08	-0.02	0.07	-0.02
	(0.01)	(0.01)	(0.17)	(0.02)	(0.10)	(0.01)
Health spending (%GDP)	-0.05	-0.04	-0.03	-0.04	-0.04	-0.03
	(0.04)	(0.04)	(0.05)	(0.05)	(0.03)	(0.03)
World Bank income group	0.19	0.18	-0.02	-0.05	-0.01	0.05
	(0.12)	(0.11)	(0.02)	(0.17)	(0.01)	(0.10)
Log of disasters	-0.09+	-0.09+	-0.05	-0.02	-0.10*	-0.10*
	(0.05)	(0.05)	(0.08)	(0.08)	(0.05)	(0.05)
Instances of war	0.18	0.18	0.45*	0.45*	0.31*	0.31*
	(0.20)	(0.20)	(0.22)	(0.22)	(0.14)	(0.14)
Ethnolinguistic fractionalization	-0.25	-0.27	0.07	0.03	-0.25	-0.27
	(0.22)	(0.21)	(0.26)	(0.24)	(0.18)	(0.18)
Political imprisonment	0.12+	0.12+	0.13	0.11	0.10*	0.10+
	(0.06)	(0.07)	(0.09)	(0.09)	(0.05)	(0.05)
Government effectiveness	-0.02	-0.08	0.45+	0.34	0.10	0.05
	(0.19)	(0.19)	(0.24)	(0.25)	(0.13)	(0.13)
Constant	0.52	0.58	1.61	0.11	1.93+	1.94
	(1.37)	(1.53)	(1.91)	(1.80)	(1.09)	(1.22)
Number of observations	117	117	118	118	131	131
Breusch-Pagan chibar2(01)		20.08*		25.34*		25.71*
		0.00		0.00		0.00
Hausman chi2(13)		8.75*		14.27*		4.36*
		0.00		0.00		0.00

+p < 0.10, *p < 0.05.

^b^Models 1 and 2 pertain to mental hospital beds, 3 and 4 to general hospital beds, and 5 and 6 to overall bed rates. Robust standard errors are in parentheses. ‘Late adopters’ is the reference category against which innovators and non-adopters were compared to. It is a combination of early adopters, early majority, and late majority in Table 4.

The results indicate support for our question that stresses a difference in bed rates by mental health policy adoption phase. Hypothesis 1 posited that late adopters of mental health policy are more likely than early adopters to reduce the number of psychiatric beds in their country. The mental hospital bed rates (model 2) and overall bed rates (model 6), shown in Table 5, confirm this hypothesis. We find no evidence in support of the corollary, or hypothesis 2, that late adopters of mental health policy have a lower likelihood of reducing the number of psychiatric beds than early adopters. In model 6, the expected rate of all psychiatric beds per 100,000 persons is 197% higher for innovators than late adopters, all else being equal. Moreover, in model 2 the expected rate for mental hospital beds per 100,000 persons also increased by a dramatic 241% for innovators as compared to late adopters, with all else being equal. These expected differences suggest late adopters are more likely to decrease the overall rate of psychiatric beds, and specifically mental hospital beds, than early adopters^q^. This was not the case for general hospital beds (model 4). Compared to late adopters, non-adopters have a pronounced 248% increase in the expected rate of general hospital beds per 100,000 population, as per model 4. This is preliminary evidence suggesting late adopters are more likely than non-adopters to cut down on the number of beds in general hospitals.

Results for the mental health system and country demographic variables offer limited support for a model emphasizing change in psychiatric bed rates. The findings indicate that a mental health law makes countries significantly more likely to decrease the rate of all psychiatric beds. This confirms previous findings that the passage of psychiatric legislation augmented augmented deinstitutionalization in countries such as Australia [158], United Kingdom [159], Italy [160], and United States [161]. The mental health workforce seemed to be a countervailing force to deinstitutionalization in that the rate of psychiatrists or nurses is directly proportional to the rates of mental hospital, general hospital, and overall psychiatric bed rates, holding all other explanatory variables constant. For every 10% increase in the rate of psychiatrists per 100,000 population, there is an expected increase of 2.24% to 2.56% in rate of psychiatric bed per 100,000 population, depending on the model. Likewise, the rate of psychiatric bed per 100,000 population is expected to increase anywhere from 2.25% to 2.56%, depending on the model, when the rate of nurses per 100,000 population increases by 10%. These independent main effects correspond to a related, long-standing clash between proponents of institutional psychiatry and advocates of mental patients’ rights [162, 163]. Psychiatric institutions may be significant contributors to the local economies of isolated communities, as is the case in former Soviet Republics, which means closing or downsizing them would dim the employment prospects of staff and instigate other negative consequences on the local economy [88, 164]. Deinstitutionalization poses as a radical challenge to the basic tenets of medicine and traditional configurations of biomedical institutions, so it is not surprising that some of its fiercest opponents are psychiatrists and nurses.

And finally, the pattern of statistical significance seemed to be especially acute when there are exogenous shocks to the mental health system in the form of war, natural disasters, and infringement of human rights. Oftentimes, mental health comes to the attention of local policymakers after a terrible global tragedy, such as the Asian Tsunami or war in Afghanistan. For every occurrence of natural disaster historically, there is an expected decrease of 1% in rate of mental hospital (model 2) or overall (model 6) psychiatric beds per 100,000 population. Disasters bring to the fore a combination of challenges -some unique to the health sector- that contribute to inequities in accessing mental health care: stigmatization, lack of empowerment within a highly vulnerable population, abuse of human rights and reluctance to change historical allocations of resources [165]. The relationship is the opposite for wars: general hospital beds per 100,000 persons is expected to increase by 57% (model 4) and overall hospital beds per 100,000 persons by 36% (model 6) for every instance of war. Nonetheless, wars help cast a spotlight on deinstitutionalization-related challenges and the opportunities to alleviate mental health problems. It is promising to find that humanitarian and emergency relief have left an imprint on affected countries such that governments have been compelled to strengthen health systems during their country’s recovery period.

The extent to which deinstitutionalization efforts is tailored to available national resources or population needs is limited. The results for the control variables show no support for the argument that population status (e.g. ethnolinguistic fractionalization, burden of MNS disorders), spending on health, or national income level changed psychiatric bed rates over the course of a decade^r^. The interaction of mental health spending and national income deserve attention in future studies, perhaps using other indicators. Even where there is a political responsiveness to the burden of mental illness, the level of available resources earmarked to address it would depend on the state of the economy. So, even if a considerable percentage of the total health budget is allocated to mental health, this would not amount to much in terms of net resources if the level of national income is low. Governments have an imperative to keep public finance under control or to make loan repayments, which means that mental health services are particularly vulnerable when public services have to be cut. Building a revenue collection and financing system that relies less on out-of-pocket payments and more on tax-funded mental health treatment or social insurance prepayment schemes is one way to advance the deinstitutionalization movement [166].

Neither civil society (e.g. NGOs, user and family association) participation nor health information technology is a significant predictor of logged bed rates. This is contrary to our prior expectations since civil society plays a vital role in challenging the prudence of government action, and they help compensate for areas where mental health is given a low priority [167, 168]. Also surprising is the non-significance of health information technology. Even if policymakers give greater priority to mental health, a paucity of information and data infrastructure are key constraints on the development of mental health services and resource allocation. And lastly, we found no evidence to support the arguments that government effectiveness, community-based care, and mental health care in primary settings affect psychiatric bed rates.

We conducted three additional analyses to check for robustness of results presented in Table 5 using alternate estimation methods. First, we estimated the three types of logged bed rates using ordinary least squares (OLS). Coefficient estimates for mental health policy adoption had magnitude and direction consonant with those produced by OLS with RE, but the OLS set did not reach significance. This is likely due to violation of key OLS assumptions. The results of Breusch-Pagan test, found on the bottom of Table 5, suggest that the OLS with RE is more appropriate than OLS alone. Second, we analyzed the same dataset using OLS with FE instead of RE. The point estimates and standard errors of Table 5 held. We suspect that country FE relevant to bed rate changes changed over a ten-year period. The Hausman test results, also found on the bottom of Table 5, pointed to the selection of RE models as the more conservative choice. In the third robustness check, we estimated logged bed rate changes with predictor variables lagged by one year. The coefficient estimates for the hypothesized effects followed the same pattern of significance reported in Table 5, except for changes in standard errors for the covariates. This suggests little autocorrelation among the three waves of the WHO *Mental Health Atlas* dataset.

## Discussion

This study contributes to the empirical literature on global health, neo-institutional theory, and governance. Deinstitutionalization represents a neo-liberal mode of emancipating people with MNS disorders from psychiatric institutions, and supporting their livelihood in the community. The impetus is to move severely mentally ill people out of psychiatric institutions and into the community, then closing down part or all of those institutions. Today, more than a half century after the first country ratified a mental health policy, neither the sentiment nor the programmatic need for deinstitutionalization has changed.

National governments reflect, enact, and propagate deinstitutionalization in varying degrees. Policies are not only artifacts of nation-states’ sovereignty, but they are also signs of support for internationally sanctioned ideologies. The act of adopting a policy allows countries to (re)build their public image and, indirectly, maintain their regional presence [163, 169]. However, national governments may not be compelled to address the needs of people with severe and chronic mental illness unless they realize the epidemic has a direct impact on the economy [136, 170, 171]. An example of the low policy priority that is given to mental health is the World Bank’s 1993 *World Development Report*, which highlighted mental disorders as a major contributor to the global burden of disease, but failed to include anything in its recommendations that would address mental health [172]. If programs associated with deinstitutionalization are to be improved, the original decisions behind enacting mental health policy must be rigorously evaluated^s^. Our study is one of the first to test whether the universal aspiration to deinstitutionalize psychiatry has been attained using empirical data on national mental health systems. Having layered a temporal dimension onto the spatial dimension of the phenomenon of policy diffusion, we were able to observe whether governments adopted or abandoned this particular core belief animating mental health policies.

We compared 193 countries belonging to different phases of mental health policy adoption on the extent to which they comply with international norms surrounding deinstitutionalization. The cornerstone of deinstitutionalization is the reduction of inpatient, psychiatric beds. We chose this particular indicator as the outcome of interest because it is an explicit, rationalized, and differentiated feature most commonly used to compare national health systems. Regression modeling of standardized rates of mental hospital, general hospital, and overall inpatient psychiatric beds revealed dramatic variations among countries in the timing and intensity of deinstitutionalization. Early adopters offer prescriptive actions that are substantiated by efficiency logics, scientific evidence (e.g. epidemiology, cost-effective analysis), and technical know-how that would not only facilitate policy diffusion, but help decision makers in later adopting countries discern appropriate from inappropriate activities and goals. Late adopters draw on earlier adopters’ experiences, and acted quickly in downscaling psychiatric institutions. Policy development and oversight are strongly linked in this scenario. And we found evidence supporting claims based on this scenario, in terms of an increase in logged rates of psychiatric beds in mental hospitals and across all biomedical institutions for innovators relative to late adopters, after adjusting for characteristics of the mental health system and the country. These results are not surprising considering that deinstitutionalization has been happening in innovator countries for the past half century, and that their psychiatric bed rates have fluctuated since inception. The results also correspond to the trans-and re-institutionalization movements, discussed in the Method section, where trends of increasing psychiatric beds and mental health wards have been documented in developed countries [95, 111, 113, 173].

Governments are just as likely to gain acceptance for unfamiliar practices, forms, and values associated with deinstitutionalization under the logic of legitimacy. There are outliers like Japan, which adopted a mental health policy as early as 1950, but also has one of the highest ratios of psychiatric beds per capita in the world [71, 174]. Policy development and oversight are decoupled in this scenario. We did not find evidence supporting this diametrically opposing argument in rates of psychiatric beds for non-adopters relative to innovators, holding other control variables constant. The evidence suggests that late adopters of mental health policy -*ceteris paribus*- are more likely than innovators to reduce psychiatric beds, but this input-output-outcome relationship merits further research attention^t^.

Our empirical results provided support for the independent impact of mental health law, workforce, disasters, war, and political imprisonment on changes in bed rates. Our analysis, however, provided no support for the integration of mental health in primary care and community-based settings, civil society participation, health information technology usage, spending on health, national income level, and population mental health and social status. The absence of supporting evidence on these variables invites more research. Alternative indicators could be developed and used in multi-level analysis to see if the results reported in this article are affected by the state of knowledge on these variables chosen or by measurement error in the indicators themselves.

Our study is limited in three ways. First, this is a study of contemporary mental health care. Our panel includes only three waves of data spanning 2001 to 2011, which prevented us from observing the dynamic process of mental health policy implementation outside of the study period. This limitation made it so that we could only make coarse grain comparison of the implementation patterns among three phases in the mental health policy diffusion cycle. It may be the case that certain factors operate well before or well beyond the horizon of the study period. One plausible scenario is that the ‘least disabled’ and ‘most independent’ patients are discharged first to show encouraging signs of moving people from hospital to community. This success would be harder to replicate with patients with higher needs and severe and chronic mental illness. As this closure process is underway, decision makers might be alarmed by the escalating costs incurred from the hospitals and from care provided in the community. Governments that do not have separate plans and funds would find it difficult to sustain both institution- and community-based services during the transition period of closing and downsizing psychiatric institutions. Deinstitutionalization could have not happened at all, fallen short at the first stage of policy adoption, reached maturity at the second stage of mental health systems reorganization, or, equally plausible, took longer than a decade to be actualized. Future research may investigate the linearity of deinstitutionalization process in different countries.

A second limitation is that the data did not allow for analysis of different translations of mental health policy, even if one was ratified. Every country has an amalgam of mental health policy components and, moreover, psychiatric beds make up just one metric of accomplishing deinstitutionalization^u^. Savvy policymakers may be tempted to concentrate on changing only the areas of mental health systems that can generate visible and immediate benefits even if the need for them is low. It is easier and cheaper to transform infrastructure rather than apply tacit knowledge in other ways, and if this is the policymakers’ underlying intent, then it would not be surprising to see more rapid reduction in beds in late-adopting countries rather than in innovator countries^v^. Tacit knowledge takes longer to gain traction, especially in countries with a decentralized government, because it is acquired mostly through learning by doing [175]. Return on workforce development investments, for example, may take several years before performance changes are observed. The (re)configuration of existing services also does not necessarily mean that there will be immediate improvements in clinical outcomes and quality of life for patients. International organizations, civil society, and others have advocated, and continue to advocate, for a long-term commitment to service delivery from governments.

Even though the crux of the philosophy of deinstitutionalization is about bridging the gap between the closure and mental hospitals and building up of community-based services, a second equally, if not more important gap that deserves mention is one that exists between the epidemiological burden of MNS disorders and the provision of psychiatric and mental health services [176]. Documented rates of psychiatric beds found in general hospitals are the closest proxy of community-based services in this study. The utilization of psychiatric beds in general hospitals has the added benefits of reducing stigma of mental disorders, facilitating public access, minimizing violations of human rights, and bringing greater attention to the diagnosis and treatment of comorbid conditions [85, 109, 177, 178]. Nonetheless, the extent to which psychiatric deinstitutionalization is embedded in cognitive and cultural frames, rules, and routines of community-based institutions remains to be measured.

Deinstitutionalization may denote reduced bed capacity, but not reduced patient demand for treatment. Aside from measuring the shift away from dependence on psychiatric institutions, the methods of this study can be replicated for changes observed in services offered by psychiatric departments in general hospitals, clinics, nursing homes, and private practitioners. It can also be replicated for parameters of mental health service utilization, such as admission, bed occupancy, readmission and relapse, default, outpatient attendance rates, and average length of stay. Deinstitutionalized mental health care also entails growing community-based services, which can be measured via the density of supportive housing, satisfaction of family caretakers, and prison populations. The extent of decoupling in different loci of health and human services prompts future studies to look at the concordance between development of community services and reduction in institution-based services.

Finally, the implementation of mental health policy depends on many country- and health system-level factors, which undoubtedly play a role in national government’s decision to have adopted it in the first place [179]. Our results indicated that bed rates changed in contingent ways, yet the control variables we used are insufficient in explaining the inter- and intra-country variance. These findings provide impetus for future study on the institutionalization of deinstitutionalizing mental health care as a process shaped by characteristics of the countries, as well as one determined by the diffusion of mental health policy internationally.

## Conclusion

In the past decades many countries have initiated extensive mental health care system reforms, and the main goal of these reforms is to transfer curative treatment for the mentally ill from psychiatric hospitals to the community. In many countries, structural reforms have been guided by mental health policies. But mental health policies are not, in and of themselves, necessarily “good”; the true measure of national health governance lies in the configuration and performance of mental health systems. Institutional theorists have argued that practices are adopted solely for symbolic reasons if the legitimacy they confer are “decoupled” from routine, technical activities of the organization [1, 54]. Yet an equally plausible claim is that institutionalized forms of practice evolved from technical forms of practice [58, 180].

Public health studies have demonstrated that community treatment models are more effective than hospital treatment models in that they can reduce the number of relapses and hospital admissions and shorten average length of stay [181–183]. Close monitoring of patient status and their adherence to treatment have also been demonstrated as effective ways to help people with serious mental illness integrate into the community [184, 185]. However, alongside the research supporting these reforms is research showing the negative, often unintended, consequences of deinstitutionalization based on outcomes such as increase in the mortality or “Revolving Door Syndrome” [95, 96, 186, 187]. Psychiatric care is not divorced from other spheres of medical and social services, and therefore sound health service planning requires cooperation among constituents and sectors in order to adequately address the global burden of MNS disorders.

## Endnotes

^a^The terms ‘institutionalization’ and ‘deinstitutionalization’ have specific denotations in different analytical communities. According to Selznick ([2]:17), institutionalization is a process by which structures or activities become “infused with value beyond the technical requirements at hand”. The phenomenon of institutionalization can be observed as “the emergence of orderly, stable, socially integrating patterns out of unstable, loosely organized, or narrowly technical activities” ([188]:238). We adopt this particular, theoretical meaning of institutionalization. The meaning of deinstitutionalization is one we invoke from the public health literature: the practice of caring for individuals in the community rather than in an institutional environment, with resultant effects on the individual patient, the individual’s family, the community, and the healthcare system [189, 190]. This is related to the definition of deinstitutionalization found in the organization sociology literature, or the erosion or discontinuity of an institutionalized organizational activity or practice, but not one we refer to directly in this article [191, 192]. In summary, in this study institutionalization entails the integration of deinstitutionalization practices and structures into existing sources such as policy and law, professional development, and industry norms.

^b^As an extension of this statement, early adopters do not have a strong reason to broadcast pilot information unless the international community sanctioned their choices in the first place [193].

^c^We would expect changes in the mix of mental health care facilities as well; specifically an increase in outpatient and day treatment facilities and a decrease in community-based psychiatric inpatient facilities, community residential facilities, and mental hospitals. We did not, however, examine these outcomes due to data quality.

^d^Legitimacy can be garnered from a vast array of sources external or internal to the organization. External sources include licensing boards, credential bodies, accreditation bodies, funding agencies, epistemic communities, professional associations, unions, rating agencies, business consortiums, public opinion polls, and the media. And internal sources include workers, managers, human resource specialists, and board members. These sources could operate independently or jointly to rate the legitimacy of a given organization.

^e^The WHO Pyramid Framework advocates for the most numerous services to be offered by informal community mental health organizations, followed by primary care settings, general hospitals, formal community mental health organizations, and lastly specialist mental health services [79].

^f^Torrey [82] remarked on how most of those who were deinstitutionalized from public psychiatric hospitals in the United States were severely mentally ill. Between 50 and 60 percent of those discharged were diagnosed with schizophrenia, another 10 to 15 percent were diagnosed with manic-depressive illness and severe depression, and an additional 10 to 15 percent were diagnosed with organic brain diseases –epilepsy, stroke, Alzheimer's disease, and brain damage secondary to trauma. The remaining individuals residing in public psychiatric hospitals had conditions such as mental retardation with psychosis, autism and other psychiatric disorders of childhood, and alcoholism and drug addiction with concurrent brain damage.

^g^Community mental health services constitute the foundation of the mental health system; this category encompasses case management, forensic community outreach teams, home treatment, rehabilitation, crisis resolution, court diversion schemes, hostels, psychiatric rehabilitation villages in rural areas, Assertive Community Treatment, and other ancillary services. Outpatient clinics have a triage function of assessing patient condition, referring patients to specialists if so needed, and providing follow-up care. And finally, inpatient care, in the form of psychiatric emergency services or short-term hospitalization, is in place to prevent long-term institutional placement. Inpatient care settings provide vigorous treatment and monitoring during acute episodes, thus allowing for continuing care in other settings between episodes.

^h^Community-based services include, but are not limited to, vocational training, supported employment, family care-giving, psychiatric beds outside mental hospitals (e.g. in general hospitals), day care services, residential care in the community, mobile clinics, outreach services, self-help and user groups, and mental health services delivered electronically.

^i^Inter-sectorial collaboration is not mentioned but is equally, if not more, important in mental health care. Full social participation for people with MNS disorders requires sustained access to jobs, schools, and other services; this requires cooperation among education, social services, labor, and justice sectors. Also outside the purview of this study are providers of therapeutic interventions outside of biomedical institutions, such as shamans, traditional healers, and priests.

^j^The count and rate of five types of mental health facilities would also be suitable candidates, but they were collected for the 2011 wave only. The types of facilities are outpatient facilities, day treatment facilities, community-based psychiatric inpatient facilities, community residential facilities, and mental hospitals.

^k^The quality of bed management depends on the availability and usage of concomitant resources available, such as home assessment, clinical gatekeepers for admissions, clear records of each admission, mental health team continual assessment, inpatient case meetings, and immediate transfer to housing upon discharge. Psychiatric beds should be prioritized for seriously mentally ill patients, variously defined as those who have had multiple admissions in the past, been legally detained, and/or failed to adhere to treatment and prescribed regimens.

^l^The year of initial adoption of national mental health policy was compiled based on a triangulation of Atlas, WHO-AIMS, and MiNDbank. Data for WHO-AIMS were collected by a team led by a focal point in each respondent country and were, in most cases, approved by its Ministry of Health [116]. MiNDbank features historical mental health policies, plans, strategies, and legislation, along with international and regional treaties, for 150 countries [118].

^m^The five components of mental health policy assessed are advocacy, promotion, prevention, treatment, and rehabilitation.

^n^A national mental health plan describes the course of action. It also indicates what has to be done, who has to do it, during what time frame and with what resources. A mental health law lays out the recriminations for the failure to carry out the terms of the plan.

^o^Public financing for health is generally derived from taxation, government-owned insurance schemes, for-profit and non-profit donors, and grants.

^p^This overlaps with seven conditions the WHO has identified as priority conditions. They are depression, schizophrenia and other psychotic disorders, suicide, epilepsy, dementia, disorders due to use of alcohol, disorders due to use of illicit drugs, and mental disorders in children. They were identified as priority conditions on the basis that they represent a high burden of mortality, morbidity, and disability; incurred large economic costs; or were associated with violations of human rights. The WHO mhGAP initiative has come up with an integrated package of interventions for each condition [143].

^q^It also deserves mention, though not reported in the tables, that the expected log mental hospital bed rates for countries with mental health policy have a 0.17 lower probability (p = 0.004) than non-adopting countries. The same relationship is observed for general hospital bed rates, though lower in magnitude and it did not reach significance (−0.017; p-value-0.902). Overall bed rates did not reach significance and displayed a coefficient in the opposite direction (0.04; p-value = 0.744).

^r^In robustness checks not shown, we created dummy variables for the four national income categories, re-ran the multivariable models with them, and made side-by-side comparisons of the results from using the ordinal or nominal variable. We found statistically significant differences for low income (p <0.10) and lower-middle income (p <0.05) versus countries of other income status when it comes to mental hospital bed rate changes. We found statistically significant difference for general hospital bed rate changes in countries belonging to upper-middle income (p <0.10) and upper income (p <0.10) relative to countries of other income status. And finally, we found statistically significant difference for overall hospital bed rate changes among countries belonging to upper-middle income (p <0.05) and upper income (p <0.05) relative to countries of other income status.

^s^There is extant research focusing on providers and patients. Provider-specific studies compare hospital and community settings using cross-sectional designs, compare types of providers or service models to divert people from hospital admissions, or estimate cost-effectiveness variations among these modalities. Patient-specific studies tend to follow people and measure changes in their clinical profile and quality of life as they experience episodes of decompensation and treatment, and make the transition from the residence in psychiatric facilities to the community.

^t^Resources are bundles of inputs used to promote health, combining staff, monetary capital, medications and other consumables. Outputs are volumes and qualities of prevention, treatment, care and rehabilitation services yielded. Outcomes are gauged in terms of symptom alleviation, changes in behavioral patterns, personal and social functioning, improved quality of life (including for families), and perhaps some wider social consequences to each individual service user.

^u^We did not use components of mental health policy as the main predictor variables because the Atlas dataset contains information for them in the 2005 wave only. The release of WHO MiNDbank would infuse data filling this information void.

^v^The five countries in the innovator category are Argentina, Czech Republic, Japan, Kuwait, and Singapore.

## Authors’ information

Gordon C. Shen (GS) is a postdoctoral fellow at Yale School of Public Health. He received his PhD in Health Services and Policy Analysis from the University of California, Berkeley, and his S.M in Epidemiology from the Harvard School of Public Health. His research spans the international relations, organization sociology, and global health domains.

Lonnie R. Snowden (LS) is a Professor of the Graduate School at the University of California, Berkeley. His research focuses on the organization and financing of health and mental health service systems, and on access and effectiveness of care to minority and underserved populations.

## Electronic supplementary material

Additional file 1: **Summary of measures.** (DOC 54 KB)

## References

[1] Meyer JW, Rowan B (1977). Institutionalized organizations: Formal structure as myth and ceremony. Am J Sociol.

[2] Selznick P (1996). Institutionalism ‘Old’ and ‘New’. Adm Sci Q.

[3] Tolbert PS, Zucker LG (1983). Institutional sources of change in the formal structure of organizations: The diffusion of civil service reform, 1880–1935. Adm Sci Q.

[4] Zucker LG (1977). The role of institutionalization in cultural persistence. Am Sociol Rev.

[5] Abrahamson E (1991). Managerial fads and fashions: the diffusion and rejection of innovations. Acad Manag Rev.

[6] Haveman HA, Russo MV, Meyer AD (2001). Organizational environments in flux: the impact of regulatory punctuations on organizational domains, CEO succession, and performance. Organ Sci.

[7] United Nations (1991). The protection of person with mental illness and the improvement of mental health care.

[8] World Health Organization (2001). The World Health Report 2001: Mental Health: New Understanding, New Hope.

[9] Jacob KS, Sharan P, Mirza I, Garrido-Cumbrera M, Seedat S, Mari JJ, Sreenivas V, Saxena S (2007). Mental health systems in countries: where are we now?. Lancet.

[10] Provan KG, Milward HB (1995). A preliminary theory of interorganizational network effectiveness: a comparative study of four community mental health systems. Adm Sci Q.

[11] Scott WR, Black BL (1986). The Organization of Mental Health Services: Societal and Community Systems, Volume 78.

[12] Rogers EM (2003). The Diffusion of Innovations.

[13] Zucker LG, Bacharach SB (1983). Organizations as Institutions. Research in the Sociology of Organizations.

[14] Braun D, Gilardi F (2006). Taking ‘Galton’s Problem’ seriously towards a theory of policy diffusion. J Theor Polit.

[15] Finnemore M, Sikkink K (1998). International norm dynamics and political change. Int Organ.

[16] Ikenberry JG (1990). The International Spread of Privatization Policies: Inducements, Learning and Policy Bandwagoning. The Political Economy of Public Sector Reform and Privatization.

[17] Clark J (1985). Policy diffusion and program scope: research directions. Publius.

[18] Goldsmith JL, Posner EA (1999). A theory of customary international law. Univ Chic Law Rev.

[19] Goldsmith J, Posner E (2002). Moral and legal rhetoric in international relations: a rational choice perspective. J Legal Stud.

[20] Waltz KN (2010). Theory of International Politics.

[21] Lindblom CE (1959). The science of muddling through. Public Adm Rev.

[22] Mukand S, Rodrik D (2002). In Search of the Holy Grail: Policy Convergence, Experimentation, and Economic Performance. No. w9134.

[23] Berk RA, Boruch RF, Chambers DL, Rossi PH, Witte AD (1985). Social policy experimentation a position paper. Eval Rev.

[24] Greenberg DH, Linksz D, Mandell M (2003). Social Experimentation and Public Policymaking.

[25] Bernanke BS (1983). Nonmonetary effects of the financial crisis in the propagation of the great depression. Am Econ Rev.

[26] Arrow KJ, Fisher AC (1974). Environmental preservation, uncertainty, and irreversibility. Q J Econ.

[27] Heclo H (1974). Modern Social Policy in Britain and Sweden: From Relief to Income Maintenance.

[28] Mansfield E (1971). Technological Change: An Introduction to a Vital Area of Modern Economics.

[29] Pindyck RS (1991). Irreversibility, uncertainty, and investment. J Econ Lit.

[30] Nelson R (1993). National Innovation Systems: A Comparative Analysis.

[31] Volden C (2006). States as policy laboratories: Emulating success in the children’s health insurance program. Am J Polit Sci.

[32] Sturm R (1999). What type of information is needed to inform mental health policy?. J Ment Health Policy Econ.

[33] Saraceno B, Saxena S (2004). Bridging the mental health research gap in low‒and middle‒income countries. Acta Psychiatr Scand.

[34] Saxena S, Paraje G, Sharan P, Karam G, Sadana R (2006). The 10/90 divide in mental health research: trends over a 10-year period. Br J Psychiatry.

[35] Meseguer C (2004). What role for learning? The diffusion of privatisation in OECD and Latin American countries. J Public Policy.

[36] Simmons B, Elkins Z (2004). The globalization of liberalization: policy diffusion in the international political economy. Am Polit Sci Rev.

[37] Haas EB (1980). Why collaborate? Issue-linkage and international regimes. World Politics.

[38] Walker JL (1969). The diffusion of innovations among the American states. Am Polit Sci Rev.

[39] Hall PA (1993). Policy paradigms, social learning, and the state: the case of economic policymaking in Britain. Comparative Politics.

[40] Berger PL, Luckmann T (1991). The Social Construction of Reality: A Treatise in the Sociology of Knowledge. No. 10.

[41] Pfeffer J, Salancik G (1978). The External Control of Organizations.

[42] March JG, Olsen JP (1998). The institutional dynamics of international political orders. Int Organ.

[43] Strang D, Meyer JW (1993). Institutional conditions for diffusion. Theory Soc.

[44] DiMaggio PJ, Powell WW (1983). The iron cage revisited institutional isomorphism and collective rationality in organizational fields. Am Sociol Rev.

[45] Meyer JW, Boli J, Thomas GM, Thomas G, Meyer J, Boli J (1987). Ontology and Rationality in the Western Cultural Account. Institutional Structure: Constituting State, Society, and the Individual.

[46] Meyer JW, Boli J, Thomas GM, Ramirez FO (1997). World society and the nation-state. Am J Sociol.

[47] McNeely C (1995). Constructing the Nation State: International Organization and Prescriptive Action. No. 113.

[48] Thelen K (1999). Historical institutionalism in comparative politics. Annual Rev Polit Sci.

[49] Meyer JW, Scott WR, Deal TE (1981). Institutional and Technical Sources of Organizational Structure: Explaining the Structure of Educational Organizations. Organization and the Human Services.

[50] Weick K (1976). Educational organizations as loosely coupled systems. Adm Sci Q.

[51] Strang D, Chang PMY (1993). The International Labor Organization and the welfare state: institutional effects on national welfare spending, 1960–80. Int Organ.

[52] Cole WM (2005). Sovereignty relinquished? Explaining commitment to the international human rights covenants, 1966–1999. Am Sociol Rev.

[53] Hafner‒Burton EM, Tsutsui K (2005). Human rights in a globalizing world: the paradox of empty promises. Am J Sociol.

[54] Burns LR, Wholey DR (1993). Adoption and abandonment of matrix management programs: Effects of organizational characteristics and interorganizational networks. Acad Manag J.

[55] Eyestone R (1977). Confusion, diffusion, and innovation. Am Polit Sci Rev.

[56] Baron JN, Dobbin FR, Jennings PD (1986). War and peace: the evolution of modern personnel administration in US industry. Am J Sociol.

[57] Westphal JD, Zajac EJ (1994). Substance and symbolism in CEOs’ long-term incentive plans. Adm Sci Q.

[58] Westphal JD, Gulati R, Shortell SM (1997). Customization or conformity? An institutional and network perspective on consequences of TQM adoption. Adm Sci Q.

[59] D’Aunno T, Sutton RI, Price RH (1991). Isomorphism and external support in conflicting institutional environments: A study of drug abuse treatment units. Acad Manag J.

[60] Edelman LB (1992). Legal ambiguity and symbolic structures: organizational mediation of civil rights law. Am J Sociol.

[61] Cyert RM, March JG (1963). A Behavioral Theory of the Firm.

[62] Abrahamson E, Rosenkopf L (1993). Institutional and competitive bandwagons: using mathematical modeling as a tool to explore innovation diffusion. Acad Manag Rev.

[63] Burt RS (1987). Social contagion and innovation: cohesion versus structural equivalence. Am J Sociol.

[64] Coleman JS, Katz E, Menzel H (1966). Medical Innovation: A Diffusion Study.

[65] Fligstein N (1985). The spread of the multidivisional form among large firms, 1919–1979. Am Sociol Rev.

[66] Elsbach KD (1994). Managing organizational legitimacy in the California cattle industry: the construction and effectiveness of verbal accounts. Adm Sci Q.

[67] Meyer JW, Scott WR (1983). Organizational Environments: Ritual and Rationality.

[68] Perrow C (1985). Overboard with myth and symbols. Am J Sociol.

[69] Scott WR, Meyer JW, Levin HM, James T (1983). Environmental Linkages and Organizational Capacity: Public and Private Schools. Comparing Public and Private Schools.

[70] Thornicroft G, Bebbington P (1989). Deinstitutionalisation–from hospital closure to service development. Br J Psychiatry.

[71] Hatta K, Nakamura H, Usui C, Kurosawa H (2010). Utility and sufficiency of psychiatric inpatient units in general hospitals: a cross‒sectional study in Tokyo. Psychiatry Clin Neurosci.

[72] Keown P, Mercer G, Scott J (2008). Retrospective analysis of hospital episode statistics, involuntary admissions under the Mental Health Act 1983, and number of psychiatric beds in England 1996–2006. BMJ.

[73] Myklebust LH, Sorgaard KW, Bjorbekkmo S, Eisemann MR, Olstad R (2010). Time-trends in the utilization of decentralized mental health services in Norway-A natural experiment: The VELO-project. Int J Ment Heal Syst.

[74] Soule SA, Snow DA, Soule SA, Kriesi H (2008). Diffusion Processes Within and Across Movements. The Blackwell Companion to Social Movements.

[75] Taylor TL, Killaspy H, Wright C, Turton P, White S, Kallert TW, Schuster M, Cervilla JA, Brangier P, Raboch J, Kalisova L, Onchev G, Dimitrov H, Mezzina R, Wolf K, Wiersma D, Visser E, Kiejna A, Piortrowski P, Ploumpidis D, Gonidakis F, Caldas-de-Almeida K, Cardoso G, King MB (2009). A systematic review of the international published literature relating to quality of institutional care for people with longer term mental health problems. BMC Psychiatry.

[76] Bruckner TA, Scheffler RM, Shen G, Yoon J, Chisholm D, Morris J, Fulton BD, Dal Poz MA, Saxena S (2011). The mental health workforce gap in low and middle income countries: A needs-based approach. Bull World Health Organ.

[77] World Health Organization (2003). Mental Health Policy and Service Guidance Package.

[78] World Health Organization: Organization of Services for Mental Health (2003). Part of Mental Health Policy and Service Guidance Package.

[79] World Health Organization: The Optimal Mix of Services (2007). Mental Health Policy, Planning and Service Development Information Sheet, Sheet 2.

[80] Reeler AP (1992). Pathways to psychiatric care in Harare. Zimbabwe. Cent Afr J Med.

[81] Steel RM, McKay IM (2000). Pathways to psychiatric admission: a study of 100 consecutive admissions to south Glasgow acute adult psychiatric wards. Health Bulletin (Edinburgh).

[82] Torrey EF (1997). Out of the Shadows: Confronting America’s Mental Illness Crisis.

[83] Breakey WR (1996). Integrated Mental Health Services: Modern Community Psychiatry.

[84] Talbott JA (1978). The Death of the Asylum: A Critical Study of State Hospital Management, Services, and Care.

[85] Sealy P, Whitehead PC (2004). Forty years of deinstitutionalization of psychiatric services in Canada: an empirical assessment. Can J Psychiatr.

[86] Bowersox NW, Szymanski BJ, McCarthy JF (2013). Associations between psychiatric inpatient Bed supply and the prevalence of serious mental illness in veterans affairs nursing homes. Am J Public Health.

[87] Pycha R, Giupponi G, Schwitzer J, Duffy D, Conca A (2011). Italian psychiatric reform 1978: milestones for Italy and Europe in 2010?. Eur Arch Psychiatry Clin Neurosci.

[88] Mundt AP, Frančišković T, Gurovich I, Heinz A, Ignatyev Y, Ismayilov F, Kalapos MP, Krasnov V, Mihai A, Mir J, Padruchny D, Potocan M, Raboch J, Taube M, Welbel M, Priebe S (2012). Changes in the provision of institutionalized mental health care in post-communist countries. PLoS One.

[89] Hartvig P, Kjelsberg E (2009). Penrose’s law revisited: the relationship between mental institution beds, prison population and crime rate. Nord J Psychiatry.

[90] Lamb HR, Weinberger LE (1998). Persons with severe mental illness in jails and prisons: a review. Psychiatr Serv.

[91] Yoon JH, Domino ME, Norton EC, Cuddeback GS, Morrissey JP (2013). The impact of changes in psychiatric bed supply on jail use by persons with severe mental illness. J Ment Health Policy Econ.

[92] Barbato A (1998). Psychiatry in transition: outcomes of mental health policy shift in Italy. Australas Psychiatry.

[93] Lurie S (2005). Comparative mental health policy: are there lessons to be learned?. Int Rev Psychiatry.

[94] Haug HJ, Rössler W (1999). Deinstitutionalization of psychiatric patients in central Europe. Eur Arch Psychiatry Clin Neurosci.

[95] Priebe S, Badesconyi A, Fioritti A, Hansson L, Kilian R, Torres-Gonzales F, Turner T, Wiersma D (2005). Reinstitutionalisation in mental health care: comparison of data on service provision from six European countries. BMJ.

[96] Priebe S, Frottier P, Gaddini A, Kilian R, Lauber C, Martínez-Leal R, Munk-Jørgensen P, Walsh D, Wiersma D, Wright D (2008). Mental health care institutions in nine European countries, 2002 to 2006. Psychiatr Serv.

[97] Salize HJ, Schanda H, Dressing H (2008). From the hospital into the community and back again-A trend towards re-institutionalisation in mental health care?. Int Rev Psychiatry.

[98] Engel GL (1977). The need for a new medical model: a challenge for biomedicine. Science.

[99] Mechanic D, Rochefort DA (1990). Deinstitutionalization: an appraisal of reform. Annu Rev Sociol.

[100] Mahoney J (2001). Path-dependent explanations of regime change: Central America in comparative perspective. Stud Comp Int Dev.

[101] Pierson P (2000). Increasing returns, path dependence, and the study of politics. Am Poli Sci Rev.

[102] Schieber G, Maeda A (1999). Health care financing and delivery in developing countries. Health Aff.

[103] Thornicroft G, Tansella M (2006). The Mental Health Matrix: A Manual to Improve Services.

[104] Lamb HR, Bachrach LL (2001). Some perspectives on deinstitutionalization. Psychiatr Serv.

[105] World Health Organization (2001). Atlas Mental Health Resources in the World.

[106] World Health Organization (2005). Mental Health Atlas 2005.

[107] World Health Organization (2011). Mental Health Atlas 2011.

[108] Mental Health Foundation (1993). Mental Illness: The Fundamental Facts.

[109] Candiago RH, da Silva SS, Gonçalves V, Belmonte-de-Abreu P (2011). Shortage and underutilization of psychiatric beds in southern Brazil: independent data of Brazilian mental health reform. Soc Psychiatry Psychiatr Epidemiol.

[110] Johnson S (2011). Has the closure of psychiatric beds gone too far?. No BMJ.

[111] Lund C, Flisher AJ (2006). Norms for mental health services in South Africa. Soc Psychiatry Psychiatr Epidemiol.

[112] Marks IM, Connolly J, Muijen M, Audini B, McNamee G, Lawrence RE (1994). Home-based versus hospital-based care for people with serious mental illness. Br J Psychiatry.

[113] Pedersen PB, Kolstad A (2009). De-institutionalisaiont and trans-institutionsation – changing trends of inpatient care in Norwegian mental health institutions 1950–2007. Int J Ment Heal Syst.

[114] Thornicroft G, Strathdee G (1994). How many psychiatric beds?. BMJ.

[115] Tyrer P (2011). Has the closure of psychiatric beds gone too far?. Yes BMJ.

[116] World Health Organization (2005). Assessment Instrument for Mental Health Systems Version 2.2.

[117] World Health Organization (2009). Mental Health Systems in Low- and Middle-Income Countries: A WHO-AIMS Cross-National Analysis.

[118] World Health Organization: WHO MiNDbank TEST SITE (2013). More Inclusiveness Needed in Disability and Development.

[119] Townsend C, Whiteford H, Baingana F, Gulbinat W, Jenkins R, Baba A, Mak FL, Manderscheid R, Mayeya J, Minoletti A, Mubbashar MH, Khandelwal S, Schilder K, Tomov T, Deva MP (2004). The mental health policy template: domains and elements for mental health policy formulation. Int Rev Psychiatry.

[120] Faydi E, Funk M, Kleintjes S, Ofori-Atta A, Ssbunnya J, Mwanza J, Kim C, Flisher A (2011). An assessment of mental health policy in Ghana, South Africa, Uganda, and Zambia. Health Res Policy Syst.

[121] Flisher AJ, Lund C, Funk M, Banda M, Bhana A, Doku V, Drew N, Kigozi FN, Knapp M, Omar M, Petersen I, Green A (2007). Mental health policy development and implementation in four African countries. J Health Psychol.

[122] Ruef M, Scott WR (1998). A multidimensional model of organizational legitimacy: Hospital survival in changing institutional environments. Adm Sci Q.

[123] Drori GS (2003). Science in the Modern World Polity: Institutionalization and Globalization.

[124] Stone D (2002). Policy Paradox: The Art of Political Decision Making, Revised ed.

[125] Anthony WA (1993). Recovery from mental illness: the guiding vision of the mental health service system in the 1990s. Psychiatr Rehabil J.

[126] Roberts G, Wolfson P (2004). The rediscovery of recovery: open to all. Adv Psychiatr Treat.

[127] Sowers W (2005). Transforming systems of care: The American Association of Community Psychiatrists guidelines for recovery oriented services. Community Ment Health J.

[128] Cobb R, Ross JK, Ross MH (1976). Agenda building as a comparative political process. Am Polit Sci Rev.

[129] Keck ME, Sikkink K (1998). Activists Beyond Borders: Advocacy Networks in International Politics.

[130] Thomas GM (1999). Constructing World Culture: Intyernational Nongovernmental Organizations Since 1875.

[131] Balla SJ (2001). Interstate professional associations and the diffusion of policy innovations. Am Polit Res.

[132] Boli J, Thomas GM (1997). World culture in the world polity: a century of international non-governmental organization. Am Sociol Rev.

[133] Gouldner AW (1957). Cosmopolitans and locals: toward an analysis of latent social roles. Adm Sci Q.

[134] Katz FE (1958). Occupational contact networks. Social Forces.

[135] Jenkins R, Baingana F, Ahmad R, McDaid D, Atun R (2011). Scaling up mental health services: where would the money come from?. Mental Health Fam Med.

[136] McDaid D, Knapp M, Raja S (2008). Barriers in the mind: promoting an economic case for mental health in low‒and middle‒income countries. World Psychiatry.

[137] Gustafson DH, Sainfort F, Eichler M, Adams L, Bisognano M, Steudel H (2003). Developing and testing a model to predict outcomes of organizational change. Health Serv Res.

[138] Salancik GR, Staw BM, Salancik GR (1977). Commitment and the Control of Organizational Behavior and Beliefs. New Directions in Organizational Behavior.

[139] Institute for Health Metrics and Evaluation (2013). Global Burden of Disease Study 2010 (GBD 2010) Results 1990–2010.

[140] Mathers CD, Fat DM, Boerma JT (2008). The Global Burden of Disease: 2004 Update.

[141] Murray CJL, Lopez AD (1997). Global mortality, disability, and the contribution of risk factors: Global Burden of Disease Study. Lancet.

[142] Whiteford HA, Degenhardt L, Rehm J, Baxter AJ, Ferrari AJ, Erskine HE, Charlson FJ, Norman RE, Flaxman AD, Johns N, Burstein R, Murray CJL, Vos T (2013). Global burden of disease attributable to mental and substance use disorders: findings from the Global Burden of Disease Study 2010. Lancet.

[143] World Health Organization (2008). mhGAP: Mental Health Action Programme: Scaling Up Care for Mental, Neurological, and Substance Use Disorders.

[144] Streveler DJ, Sherlock SM (2004). Health Management Information Systems for Resource Allocation and Purchasing in Developing Countries. Health, Nutrition and Population Discussion Paper.

[145] Hyman S, Chisholm D, Kessler R, Patel V, Whiteford H (2006). Mental Disorders. Disease Control Priorities Related to Mental, Neurological, Developmental and Substance Abuse Disorders, Chapter 31.

[146] Lund C, Tomlinson M, De Silva M, Fekadu A, Shidhaye R, Jordans M, Petersen I, Bhana A, Kigozi F, Prince M, Thornicroft G, Hanlon C, Kakuma R, McDaid D, Saxena S, Chisholm D, Raja S, Kippen-Wood S, Honikman S, Fairall L, Patel V (2012). PRIME: A programme to reduce the treatment gap for mental disorders in five low-and middle-income countries. PLoS Med.

[147] Patel V, Belkin GS, Chockalingam A, Cooper J, Saxena S, Unützer J (2013). Grand challenges: integrating mental health into priority health care platforms. PloS One.

[148] Thornicroft G, Alem A, Santos RA, Barley E, Drake RE, Gregorio G, Hanlon C, Ito H, Latimer R, Law A, Mari J, McGeorge P, Padmavati R, Razzouk D, Semrau M, Setoya Y, Thara R, Wondimagegn D (2010). WPA guidance on steps, obstacles and mistakes to avoid in the implementation of community mental health care. World Psychiatry.

[149] World Health Organization and World Organization of Family Doctors (WONCA) (2008). Integrating Mental Health into Primary Care: A Global Perspective.

[150] Frank RG, Glied SA (2006). Better but not Well: Mental Health Policy in the United States Since 1950.

[151] Bank W (2012). Atlas Method.

[152] EM-DAT (2002). The OFDA/CRED International Disaster Database.

[153] Singer JD, Small M (1972). The Wages of War, 1816–1965: A Statistical Handbook.

[154] Small M, Singer JD, Bennett R (1982). Resort to arms: International and civil wars, 1816-1980, Volume 4.

[155] Alesina A, Devleeschauwer A, Easterly W, Kurlat S, Wacziarg R (2003). Fractionalization. J Econ Growth.

[156] Cingranelli DL, Richards DL (1999). Measuring the level, pattern, and sequence of government respect for physical integrity rights. Int Stud Q.

[157] Cingranelli DL, Richards DL, Clay KC (2013). The cingranelli-richards (CIRI) human rights dataset.

[158] Callaghan S, Ryan CJ (2012). Rising to the human rights challenge in compulsory treatment–new approaches to mental health law in Australia. Aust N Z J Psychiatry.

[159] Linford S (2005). Mental health law: compulsory treatment in a general medical setting. Br J Hosp Med.

[160] Palermo GB (1991). The 1978 Italian mental health law—a personal evaluation: a review. J R Soc Med.

[161] McGarry LA, Kaplan HA (1973). Overview: current trends in mental health law. Am J Psychiatry.

[162] Koyanagi C, Henry J (2007). Learning from History. Deinstitutionalization of People With Mental Illness as Precursor to Long-Term Care Reform.

[163] Novella EJ (2010). Mental health care and the politics of inclusion: a social systems account of psychiatric deinstitutionalization. Theor Med Bioeth.

[164] Scheffler RM, Potůcĕk M (2008). Mental Health Care Reform in the Czech and Slovak Republics, 1989 to the Present.

[165] World Health Organization (2013). Building Back Better: Sustainable Mental Health Care After Emergencies.

[166] Dixon A, McDaid D, Knapp M, Curran C (2006). Financing mental health services in low-and middle-income countries. Health Policy Plan.

[167] Sanchez SH, Katz CL (2006). International disaster mental health services: a survey. Psychiatr Serv.

[168] Wright N, Stickley T (2013). Concepts of social inclusion, exclusion and mental health: a review of the international literature. J Psychiatr Ment Health Nurs.

[169] Hazelton M (2005). Mental health reform, citizenship and human rights in four countries. Health Sociol Rev.

[170] Knapp M (2012). Mental health in an age of austerity. Evid Based Ment Health.

[171] Knapp M, Beecham J, McDaid D, Matosevic T, Smith M (2011). The economic consequences of deinstitutionalisation of mental health services: lessons from a systematic review of European experience. Health Soc Care Community.

[172] Bank W (1993). World Development Report 1993: Investing in Health.

[173] Scull A (2003). The asylum as community or the community as asylum. Crime.

[174] Kuno E, Asukai M (2000). Efforts toward building a community-based mental health system in Japan. Int J Law Psychiatry.

[175] Strumpf KS (2002). Does government decentralization increase policy innovation?. J Public Econ Theory.

[176] Wang PS, Aguilar-Gaxiola S, Alonso J, Angermeyer MC, Borges G, Bromet EJ, Bruffaerts R, de Girlolamo G, de Graaf R, Gureje O, Haro JM, Karam EG, Kessler RC, Kovess V, Lane MC, Lee S, Levinson D, Ono Y, Patukhova M, Posada-Villa J, Seedat S, Wells JE (2007). Use of mental health services for anxiety, mood, and substance disorders in 17 countries in the WHO world mental health surveys. Lancet.

[177] Bauer M, Kunze H, Von Cranach M, Fritze J, Becker T (2001). Psychiatric reform in Germany. Acta Psychiatr Scand.

[178] Vázquez‒Barquero JL, Garcia J, Torres‒González F (2001). Spanish psychiatric reform: what can be learned from two decades of experience?. Acta Psychiatr Scand.

[179] Shen GC (2014). Cross-national diffusion of mental health policy. Int J Health Policy Manage.

[180] Ansari SM, Fiss PC, Zajac EJ (2010). Made to fit: how practices vary as they diffuse. Acad Manag Rev.

[181] European Commission (2013). Mental health systems in the european union member states, status of mental health in populations and benefits to be expected from investments into mental health.

[182] Stein LI, Test MA (1978). Alternatives to Mental Hospital Treatment.

[183] Tyrer P, Turner R, Johnson AL (1989). Integrated hospital and community psychiatric services and use of inpatient beds. BMJ.

[184] Davidson L, Strauss JS (1992). Sense of self in recovery from severe mental illness. Br J Med Psychol.

[185] Slade M (2010). Mental illness and well-being: the central importance of positive psychology and recovery approaches. BMC Health Serv Res.

[186] Gafoor R, Nitsch D, McCrone P, Craig TK, Garety PA, Power P, McGuire P (2010). Effect of early intervention on 5-year outcome in non-affective psychosis. Br J Psychiatry.

[187] Strauss D, Kastner TA (1996). Comparative mortality of people with mental retardation in institutions and the community. AJMR.

[188] Broom L, Selznick P (1955). Sociology: A Text with Adapted Readings.

[189] Fakhoury W, Priebe S (2002). The process of deinstitutionalization: an international overview. Curr Opin Psychiatry.

[190] Bachrach LL (1976). Deinstitutionalization: An Analytical Review and Sociological Perspective.

[191] Davis GF, Diekmann KA, Tinsley CH (1994). The decline and fall of the conglomerate firm in the 1980s: The deinstitutionalization of an organizational form. Am Sociol Rev.

[192] Oliver C (1992). The antecedents of deinstitutionalization. Organ Stud.

[193] Davis GF, Greve HR (1997). Corporate elite networks and governance changes in the 1980s. Am J Sociol.

