# Supplementary material for: Institutionalization of deinstitutionalization: a cross-national analysis of mental health system reform
Source: Int J Ment Health Syst. 2014 Nov 22;8:47. doi: 10.1186/1752-4458-8-47 (PMC4253997; doi:10.1186/1752-4458-8-47)
Supplement: Supplementary file 1 — Additional file 1: Summary of measures. (DOC 54 KB) [file 13033_2014_207_MOESM1_ESM.doc]

**Additional file 1: Summary of measures**

|  | **Indicator** | | **Definition** | **Source of Data** | **Waves** | **Operationalization** |
| --- | --- | --- | --- | --- | --- | --- |
| **Dep.** | Psychiatric beds | lnBEDS_MH_R | Rate of beds in mental hospitals per 100,000 population | WHO's Mental Health Atlas | 3 | Interval (logged rate) |
| **Indep. - H1 (Eff.); H2 (Legit.)** | Mental health policy adoption | i.MHPol_Yr_Diff | Five phases: innovators; early adopters; early majority; late majority; laggards | WHO's Mental Health Atlas; WHO-AIMS; WHO's MiNDbank | 3 | Ordinal variable constructed based on spline interpolaton of year |
| **Ctrl - M.h. system characteristics** | Mental health plan adoption | MHPlan_Yr_Diff | Five phases: innovators; early adopters; early majority; late majority; laggards | WHO's Mental Health Atlas | 3 | Ordinal variable constructed based on spline interpolaton of year |
|  | Mental health law adoption | MHLaw_Yr_Diff | Five phases: innovators; early adopters; early majority; late majority; laggards | WHO's Mental Health Atlas | 3 | Ordinal variable constructed based on spline interpolaton of year |
|  | Human resources - Psychiatrists | lnPSYCHI_R | Rate of psychiatrists per 100,000 population | WHO's Mental Health Atlas | 3 | Interval (logged rate) |
|  | Human resources - Nurses | lnNURSE_R | Rate of nurses per 100,000 population | WHO's Mental Health Atlas | 3 | Interval (logged rate) |
|  | Human resources - Psychologists | lnPSYCHO_R | Rate of psychologists per 100,000 population | WHO's Mental Health Atlas | 3 | Interval (logged rate) |
|  | Human resources - Social Workers | lnSOCWORK_R | Rate of social workers per 100,000 population | WHO's Mental Health Atlas | 3 | Interval (logged rate) |
|  | User and family associations | USERFAM | At least one user or family association in the country | WHO's Mental Health Atlas | 1 | Nominal (Y/N) |
|  | NGOs | NGO | NGOs are involved in mental health in the country | WHO's Mental Health Atlas | 2 | Nominal (Y/N) |
|  | Service integration | MHPRIM | Mental health within primary health care | WHO's Mental Health Atlas | 3 | Nominal (Y/N) |
|  | Community-based care | COMMCARE_R | System of community-based care for mental health | WHO's Mental Health Atlas | 3 | Nominal (Y/N) |
|  | Information system | HIT | Data on mental disorders in annual reporting systems | WHO's Mental Health Atlas | 3 | Nominal (Y/N) |
|  | Financing and budget | MentalHealth_GDP | Total expenditure on mental health as % of GDP | WHO's National Health Account database; World Bank's World Development Indicators | 3 | Ratio (%) |
|  | Medicine | Pharm_Health | Expenditure on pharmaceuticals as % of total expenditure on health | WHO's World Medicines Situation Survey | 1 (1999) | Ratio (%) |
|  | Disorder prevalence | lnDALY_MNS_R | Disability-Adjusted Life Years (DALY) rates per 100,000 individuals for mental, behavioral and substance use disorders | Institute for Health Metrics and Evaluation's Global Burden of Disease Study | 3: 2000 (region only); 2004 (countries); 2010 (countries) | Interval (logged rate) |
| **Ctrl - Country char.** | Income group | INCOME | Income groups (low, lower-middle, upper-middle, high), based on GNI per capita, Atlas method (current US$) | World Bank's World Development Indicators; OECD's International Development Statistics online database | 3 | Ordinal (4 income groups) |
|  | Natural disasters | lnDISASTER | Number of natural and technological disasters | Centre for Research on the Epidemiology of Disasters's EM-DAT The International Disaster Database | 3 | Interval (logged # disasters) |
|  | Man made disasters | WAR, EVENT | Number of intra-, inter- and extra-state wars | Correlates of War Project's The New COW War Data (v4.0) | 3 | Nominal (Y/N instance of war); Interval (# of wars) |
|  | Ethnolinguistic gradient | ELF: ETHNIC, LANGUAGE, RELIGION | Fractionalization Index is the probability (0-1) that two randomly drawn individuals come from the same population. It is a cumulative index of ethnic, language, and religion heterogeneity in countries. | Alberto Alesina et al. 2003 | 1 (mostly 2001) | Ratio (probability) |
|  | Human rights | PHYSINT: POLPRIS | Physical Integrity Right Index ranges from 0 "no government respect for these four rights" to 8 "full government respect for these four rights." Political imprisonment, as one dimension of physical integrity, is an ordinal variable that ranges from 0 "many people imprisoned because of their religious, political or other beliefs in a given year," 1 "few people imprisoned," 2 "no persons imprisoned" | Cingranelli-Richards (CIRI) Human Rights Dataset | 3 | Interval (scale) |
|  | Government effectiveness | GOVEFF | Government Effectiveness Index, which ranges from -2.5 "low" to 2.5"high" | World Bank's Governance Matters Project | 3 | Interval (scale) |
